# A Cost Reduction Strategy for Aluminum–Polymer Batteries: The Role of Impurities within AlCl_3_

**DOI:** 10.1021/acsomega.5c13009

**Published:** 2026-05-07

**Authors:** Mohammad Mostafizar Rahman, Amir Mohammad, Shuvrodev Biswas, Thomas Köhler, Hartmut Stöcker, Dirk C. Meyer

**Affiliations:** † Institute of Experimental Physics, 565761 TU Bergakademie Freiberg, Leipziger Str. 23, Freiberg 09599, Germany; ‡ Center for Efficient High-Temperature Material Conversion, 26545 TU Bergakademie Freiberg, Winklerstr. 5, Freiberg 09599, Germany

## Abstract

The electrolyte plays a crucial role in defining the electrochemical performance of aluminum-ion batteries (AIBs), in which AlCl_3_ serves as the primary ion source. This work systematically evaluates the influence of AlCl_3_-sourced impurities on polyamide-based solid polymer electrolytes (SPEs), with an emphasis on both electrochemical behavior and cost efficiency. SPEs are synthesized using a polyamide matrix and an AlCl_3_:Et_3_NHCl ionic liquid containing excess AlCl_3_. Six AlCl_3_ precursors with varying purities (98%–99.999%) and costs (0.028 Euro/g–6.198 Euro/g) are employed. Electrochemical analysis reveals that the AlCl_3_ purity exerts no systematic influence on key performance indicators. Reaction kinetics, aluminum stripping/plating behavior, ionic conductivity (0.19–0.21 mS cm^–1^), electrochemical stability window (2.70–2.83 V), and Coulombic efficiency (98.0–99.5%) remain nearly identical across all samples. Likewise, specific capacities (28.7–43.5 mAh g^–1^) and energy densities (3.3–4.5 Wh kg^–1^) exhibit no clear correlation with declared impurity levels (≤2%). Instead, variations arise primarily from electrolyte formulation and cell fabrication conditions. Complementary microscopic and compositional analyses reveal no significant impurity-induced morphological or compositional differences on the Al anode surface, supporting the electrochemical findings. Cost analysis indicates that low-purity AlCl_3_ (0.028 Euro/g) delivers comparable performance to high-purity salts (6.198 Euro/g), resulting in a 94% reduction in electrolyte cost and a 56% decrease in total cell cost. These findings highlight the economic viability of lower-purity AlCl_3_ for scalable AIB production.

## Graphical abstract

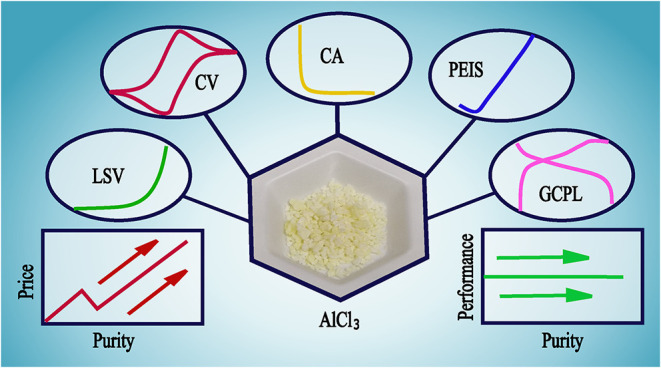

## Introduction

Aluminum-ion batteries (AIBs) have attracted increasing attention due to their low cost, high natural abundance, intrinsic safety, and high volumetric capacity compared to lithium-ion batteries (LIBs).^1–3^ Among the key battery components, the electrolyte plays a central role in governing ionic transport, electrochemical stability, and interfacial compatibility. Aluminum chloride (AlCl_3_) has been widely employed as the primary aluminum source in diverse electrolyte systems, including molten salts, organic solvents, deep eutectic solvents, ionic liquids (ILs), gels, and solid polymer electrolytes (SPEs)^4–10^ (see Supporting Figure S1). Among these systems, IL-based electrolytes have been particularly well studied. In such systems, AlCl_3_ reacts with organic salts to form AlCl_4_^–^ and Al_2_Cl_7_^–^ complex ions, which are responsible for charge transport and reversible aluminum plating/stripping.

Despite the central role of AlCl_3_, its purity is rarely discussed in the literature. Impurities present in AlCl_3_ salts can influence electrolyte performance by inducing side reactions, promoting electrode corrosion, altering ion speciation, or impeding ion mobility.^11,12^ Some inorganic impurities, such as Al_2_O_3_ and TiO_2_, may act as benign fillers or structural modifiers.^13,14^ Metal-ion impurities, including alkali and alkaline earth metals, can influence polymer chain dynamics and electrochemical behavior: at low concentrations, they may enhance ionic conductivity and mechanical flexibility,^15,16^ whereas higher levels can cause polymer plasticization and structural degradation.^17,18^ In contrast, transition metal ions (e.g., Fe^3+^, Zn^2+^) can improve SPE performance by catalyzing redox reactions, enhancing ionic conductivity, and stabilizing the polymer matrix.^19–21^ However, they may also promote electrolyte decomposition, trigger side reactions, and form inactive [*M*(AlCl_4_)_*n*_]^−^ complexes that reduce active ion concentration.^11,12^ Nonmetallic impurities, such as sulfur species, are generally detrimental, causing electrode poisoning, interfacial instability, and reduced charge-transfer efficiency, as well as promoting the formation of insulating interfacial phases, altering SEI composition, and deactivating electrochemically active sites.^22–25^ Water, a common impurity in highly hygroscopic AlCl_3_, may further react to form acidic byproducts and compromise electrochemical stability.^26^ Overall, impurity effects can be both beneficial and detrimental, yet they remain poorly understood, especially in solid polymer electrolytes for AIBs.

From an economic perspective, AIBs are inherently attractive due to the abundance and direct use of aluminum metal.^27^ However, the cost of AIB electrolytes remains a concern, largely due to the high price of organic salts and high-purity AlCl_3_. Although alternative low-cost electrolytes—such as AlCl_3_/urea, AlCl_3_/amide-based systems, and Al­[OTf]_3_-based electrolytes—have been explored,^5,6,28^ the impact of AlCl_3_ salt purity on battery performance has not been systematically investigated. Notably, AlCl_3_ prices vary substantially with purity and supplier-specific product grades, raising the question of whether lower-purity, industrial-grade salts could be used without sacrificing electrochemical performance.

Recently, a solvent-free synthesis route was reported for preparing freestanding polyamide-based SPEs by directly complexing polyamide 6 (PA6) with AlCl_3_ and Et_3_NHCl.^10^ The resulting SPE exhibited excellent mechanical integrity, strong electrode–electrolyte interfaces, and favorable electrochemical properties, including an ionic conductivity of ∼0.3 mS cm^–1^ at room temperature and stable aluminum plating/stripping. When implemented in Al/graphite cells, the electrolyte enabled stable cycling with a cathode capacity of ∼33 mAh g^–1^ and compatibility with standard stainless-steel cell housings.^10^

In this work, we extend this approach by systematically investigating the effect of AlCl_3_ salt purity on the electrochemical performance of PA6-based SPEs for AIBs. While the absolute electrochemical metrics are moderate, they are fully consistent with solvent-free SPE systems and sufficiently robust to enable a rigorous evaluation of impurity tolerance and cost scalability. Six AlCl_3_ salts with different purities and price points were used to prepare ILs and the corresponding SPEs via the same solvent-free method. Electrochemical performance was evaluated using Al/SPE/Al and Mo/SPE/Mo symmetric cells as well as Al/SPE/graphite full cells. By directly correlating salt purity with electrolyte behavior and cell performance, this study establishes the feasibility of using lower-cost AlCl_3_ salts and provides practical guidance for scalable aluminum-ion battery technologies.

## Experimental Methods

### Materials

A total of six different commercial-grade AlCl_3_ salts, with purities ranging from 98% to 99.999%, were procured from suppliers representing various quality tiers, including industrial, laboratory, and electronic-grade levels. These materials, which also varied significantly in price, were used to prepare the SPEs in order to assess the influence of precursor purity on electrochemical performance and practical feasibility. Of these, five AlCl_3_ salts were procured from Alfa Aesar, while the remaining one was obtained from Sigma-Aldrich (see Table 1). It should be noted that, despite similar nominal purities, the commercial prices of AlCl_3_ salts vary substantially due to supplier-specific product grades, impurity certification, batch consistency, packaging scale, and intended application rather than purity alone. The optical appearances of these salts are shown in Figure S2 (see Supporting Information), where salts 1 to 3 appear as fine powders, while salts 4 to 6 exhibit a granular form. Triethylamine hydrochloride (Et_3_NHCl, 99.9%), an organic salt, was acquired from Sigma-Aldrich. Polyamide (PA, 95.2%, Goodfellow) was used as the polymer host material. For the anode material, a 30 μm thick aluminum foil (≥99%) from Carl Roth GmbH + Co. KG was employed. The cathode was composed of spherical graphite (SpG, 99.95%, Sigma-Aldrich) as the active material, along with carbon black (CB, 99.99%, Alfa Aesar) as a conductivity modifier, poly­(methyl methacrylate) (PMMA, Alfa Aesar) as a binder, and *N*-methylpyrrolidone (NMP, 99%, Sigma-Aldrich) as the solvent. The current collector was a 50 μm thick conductive polyimide film purchased from Ryan Technology & Co., Ltd. A 500 μm thick and 16 mm diameter stainless-steel spacer was used in the cell assembly. The cell was enclosed in a stainless-steel case of CR2016 size.

**Table 1:** Purity of AlCl_3_ Salts, Company Name and Prices per 100 g Salt

AlCl_3_	purity (%)	Company	price/100 g (Euro)
Salt-1	99.999	Alfa Aesar	619.80
Salt-2	99.999	Alfa Aesar	586.00
Salt-3	99.99	Alfa Aesar	218.00
Salt-4	99	Alfa Aesar	47.90
Salt-5	99	Alfa Aesar	2.78
Salt-6	98	Sigma-Aldrich	36.10

### Preparation of Polymer Electrolyte

The solid polymer electrolyte (SPE) preparation procedure follows the methodology outlined in previous studies.^10,29^ In the first step, the IL was prepared by the solid-state mixing of AlCl_3_ and Et_3_NHCl in a molar ratio of 2:1. The mixture was then heated to 80 °C for 3 h to ensure complete mixing. Subsequently, the SPE was prepared by mixing the IL, excess AlCl_3_ salt, and PA (polyamide) in a molar ratio of 1:0.5:1. Excess AlCl_3_ was defined here as an additional AlCl_3_ relative to the stoichiometric amount, resulting in a total of 2.5 AlCl_3_ in the polymer system. The amount of extra AlCl_3_ added to the solid polymer electrolyte (SPE) formulation was calculated using a mass scaling approach based on a reference formulation (see Supporting Section S.1.2). The mixture was then annealed at 100 °C for 3 h to complete the polymer formation. The ILs prepared with six salts were then used for the preparation of SPEs. A total of 6 g of the SPE was prepared using each salt. The different steps of the SPE preparation process are shown in Figure S3 (see Supporting Information).

To ensure reliability and obtain statistical data for analysis, ILs were repeated four times (four batches) using different purities of the AlCl_3_ salts. A total of 24 IL samples were prepared, with each batch consisting of six samples labeled IL-11 to IL-46 (see Supporting Table S1). These ILs were subsequently utilized to prepare SPEs, which were labeled S-11 to S-46, as detailed in Table S2. For both IL and SPE samples, position 1 of the number refers to the batch, and position 2 refers to the salt sample. Figure S3A,C show only the third batch of samples (ILs and SPEs) prepared using different purities of AlCl_3_ salts.

### Impurity Testing and Physical Characterization

The impurities in various AlCl_3_ salts were analyzed by using inductively coupled plasma optical emission spectrometry (ICP-OES). The measurements were carried out by using a Thermo Scientific iCAP 6000 Series ICP-ES. The results are shown in Table 2. The physical characterization and properties of the solid polymer electrolyte have been described in detail in the literature by Mohammad et al.^10^

**Table 2:** Impurities Present in AlCl_3_ Salts in Parts per Million According to ICP–OES Measurements

element number	impurity element	Salt-1 (ppm)	Salt-2 (ppm)	Salt-3 (ppm)	Salt-4 (ppm)	Salt-5 (ppm)	Salt-6 (ppm)
11	Na		12.70				54.50
12	Mg	8.19	2.92	6.23	1.78	1.49	5.54
16	S		53.86		12.20		20.00
20	Ca	7.57	51.84	3.54	2.06		37.20
26	Fe		5.83		9.82	5.61	25.40
30	Zn		4.52		31.10	4.62	9.08
total		15.75	131.67	9.77	56.96	11.72	151.72

### Fourier Transform Infrared Spectroscopy (FTIR)

Attenuated Total Reflection Fourier Transform Infrared (ATR-FTIR) spectroscopy was employed to characterize both the six AlCl_3_ salts (98–99.999%) and the corresponding PA-based SPE films prepared from each salt. The ATR-FTIR spectra of the as-received AlCl_3_ powders and the polymer electrolytes were recorded in the range of 190–3800 cm^–1^ using a Bruker Tensor 27 spectrometer equipped with a Bruker Platinum diamond ATR unit. Measurements were performed with a spectral resolution of 2 cm^–1^ (apodized) and a beam diameter of 5 mm. A Blackman–Harris 3-term function was applied for apodization. For both the background and sample spectra, 128 scans were recorded. Prior to analysis, each sample was directly applied to the diamond ATR crystal and pressed using a constant-force plunger with a sealing ring inside an argon-filled glovebox, ensuring uniform contact and preventing air exposure.

The resulting ATR spectra were plotted as absorption spectra based on the following equation: ATR = absorption coefficient × ν/1000, where ν is the wavenumber. Since the penetration depth in ATR measurements is inversely proportional to the wavenumber, the spectra were normalized to a constant penetration depth using Bruker OPUS software.^30,31^

Vibrational bands were analyzed to assess potential spectral variations depending on salt purity. For the AlCl_3_ salts, focus was placed on the broad O–H stretching region (3000–3600 cm^–1^), and these bands were integrated to evaluate the residual moisture content. For improved comparability of the spectra of the AlCl_3_ salts, the data were normalized to the intensity of the AlCl_6_ vibrational mode. In contrast, for the SPEs, the characteristic Al–Cl stretching modes of AlCl_4_^–^ and Al_2_Cl_7_^–^, observed in the regions of 200–650 cm^–1^, were examined. To determine the Al_2_Cl_7_^–^ fraction, the spectra were fitted using pseudo-Voigt functions,^10,32^ and the relative contribution was quantified from the area ratio of the dominant AlCl_4_^–^ and Al_2_Cl_7_^–^ stretching bands (see Supporting Section S.3.1).

### Preparation of Cathode

The graphite cathode was fabricated via a conventional slurry-casting method, following previously reported procedures.^10,29^ Briefly, SpG was used as the active material with CB and PMMA as the conductive additive and binder, respectively, in a weight ratio of 80:10:10. NMP served as the solvent with a solid-to-solvent ratio of 40:60. The slurry was prepared by using 12 g of solid material in total.

For the slurry preparation, PMMA was first dissolved in NMP at 50 °C under magnetic stirring for 12 h until a clear solution was obtained. Subsequently, a dry mixture of SpG and CB was added and homogenized using a high-shear mixer (IKA T25 Ultra-Turrax) at 18,000 rpm for 40 min. The resulting slurry was then cast onto a polyimide conductive film (13 × 10 cm^2^) using a doctor blade and dried at 75 °C for 10 h. Finally, the dried electrodes were calendered to obtain a dense and uniform active layer.

### Coin Cell Construction

Three types of cells were assembled for electrochemical characterization, namely Al/SPE/Al and Mo/SPE/Mo symmetric cells, and Al/SPE/graphite full cells, following procedures reported in previous studies.^10,33^ For Al/SPE/Al cells, the solid polymer electrolyte (SPE) was sandwiched between polished (by SiC paper 500 mesh) and unpolished Al foils, followed by thermal softening at 80 °C and pressing to obtain a uniform thickness of 200 μm. After cooling to room temperature, the unpolished Al foil was removed, and the assembly was punched into 16 mm discs. The counter electrode was then fitted, and the cell was assembled into CR2016 coin cells with appropriate spacers under controlled pressure (0.6 tons). The same general procedure was applied to fabricate the other cell configurations. Details of the component arrangement and cell design are provided in Table S4 and Figure S4 (see Supporting Information). Electrochemical measurements were carried out using the respective cell configurations, as described in the relevant sections.

### Electrochemical Testing

Cyclic voltammetry (CV), chronoamperometry (CA), potentiostatic electrochemical impedance spectroscopy (PEIS), and linear sweep voltammetry (LSV) measurements were performed on Al/SPE/Al and Mo/SPE/Mo symmetric cells at room temperature using a BioLogic SP300 potentiostat. All electrochemical measurements were carried out using a two-electrode configuration in which the counter electrode also served as the reference electrode. For the CV measurements of different batches of solid polymer electrolytes (SPEs), a voltage range of −0.5 to 0.5 V was applied at a scan rate of 1 mV s^–1^. Each measurement was performed over three cycles to ensure the stability and reproducibility of the cyclic voltammograms. For CA, a constant DC potential of 0.5 V was applied, and the resulting current was recorded over time. For PEIS, a 5 mV voltage was applied over a frequency range of 0.5 Hz to 1 MHz. The LSV measurements were recorded at a scan rate of 1 mV s^–1^ in the potential range of 0.5 to 3 V.

The CV and galvanostatic cycling with potential limitation (GCPL) were carried out on full cells (Al/SPE/SpG) at room temperature using a BioLogic BCS-805 battery tester. The CV measurements were performed in a voltage range of 0 to 2.5 V with a scan rate of 1 mV s^–1^. CV measurements were taken for three cycles for each measurement. The operating charge/discharge cutoff voltage range was set from 0.5 to 2.35 V with a current density of 20 mA g^–1^ at room temperature. During the charge–discharge tests, the cells were maintained at the maximum charging voltage for 30 min before initiating the discharge cycle.

### Surface Characterization of Anode

The surface morphology of the anode surface was examined using a scanning electron microscope (SEM, FEI Helios NanoLab 600i) operated at an accelerating voltage of 10 kV. Prior to imaging, the cell was carefully disassembled, and the anode was gently cleaned using soft tissue. Elemental analysis of the anode surface was performed using energy-dispersive X-ray spectroscopy (EDX, Apollo XL SDD detector) integrated with SEM. The EDX measurements were conducted at an accelerating voltage of 20 kV, with spectra acquired from selected regions of interest on the sample surface.

## Results and Discussion

### Impurities Present and Visual Observation Analysis of IL

The impurities in all AlCl_3_ salts were analyzed using the ICP-OES technique, and the results are summarized in Table 2, which do not fully align with the data provided by the supplier (see Table S3). The analysis indicates that only a few metals, namely Na, Mg, Ca, Fe, and Zn, along with the nonmetal S, were detected as impurities. These elements can be categorized as follows: Na is an alkali metal; Mg and Ca are alkaline earth metals; Fe and Zn are transition metals; and S is a nonmetal. All metals can be present as metal chlorides, such as MgCl_2_, CaCl_2_, FeCl_3_, and similar compounds, as impurities. Additionally, water may be present as an impurity according to company sources (see Table S3), forming complex compounds such as AlCl_3_·6H_2_O or metal chloride hydrates.^34,35^ These impurities are likely introduced either through the raw materials used in the synthesis of AlCl_3_ or as a consequence of the production methods employed.

Visual inspection of the IL samples (Figure S3A,a,f) reveals that IL-31, IL-33, and IL-36 exhibit a slight yellowish tint; IL-32 and IL-35 show a moderate yellowish coloration, while the remaining sample appears clear and transparent. The yellowish coloration is attributed to impurities originating from residual water in the solution or to strongly exothermic reactions induced by it, as supported by the findings of Vaughan and Dreisinger.^36^ The intensity of the color depends on the amount of water present or on the formation of hydrated complexes.

FTIR spectroscopy was employed to qualitatively assess the water content in six AlCl_3_ salts (salt-1 to salt-6), focusing on the vibrational regions associated with moisture. The spectra of the AlCl_3_ powders, shown in Figure 1a, exhibit characteristic vibrational modes of AlCl_6_ octahedra in the far-infrared region, consistent with previously reported data.^37^ Most of the salts display a prominent O–H stretching band around 3350 cm^–1^ and a bending vibration near 1615 cm^–1^—both indicative of moisture presence. A peak near 1090 cm^–1^ is attributed to Al–OH–Al hydrated complexes, while bands observed between 560 and 760 cm^–1^ correspond to Al–OH stretching modes.^38,39^ Additionally, strong and weak features below 500 cm^–1^ are assigned to Al–Cl stretching vibrations. All vibrational modes of AlCl_3_ are listed in Table S5 (see Supporting Information).

**Figure 1:**
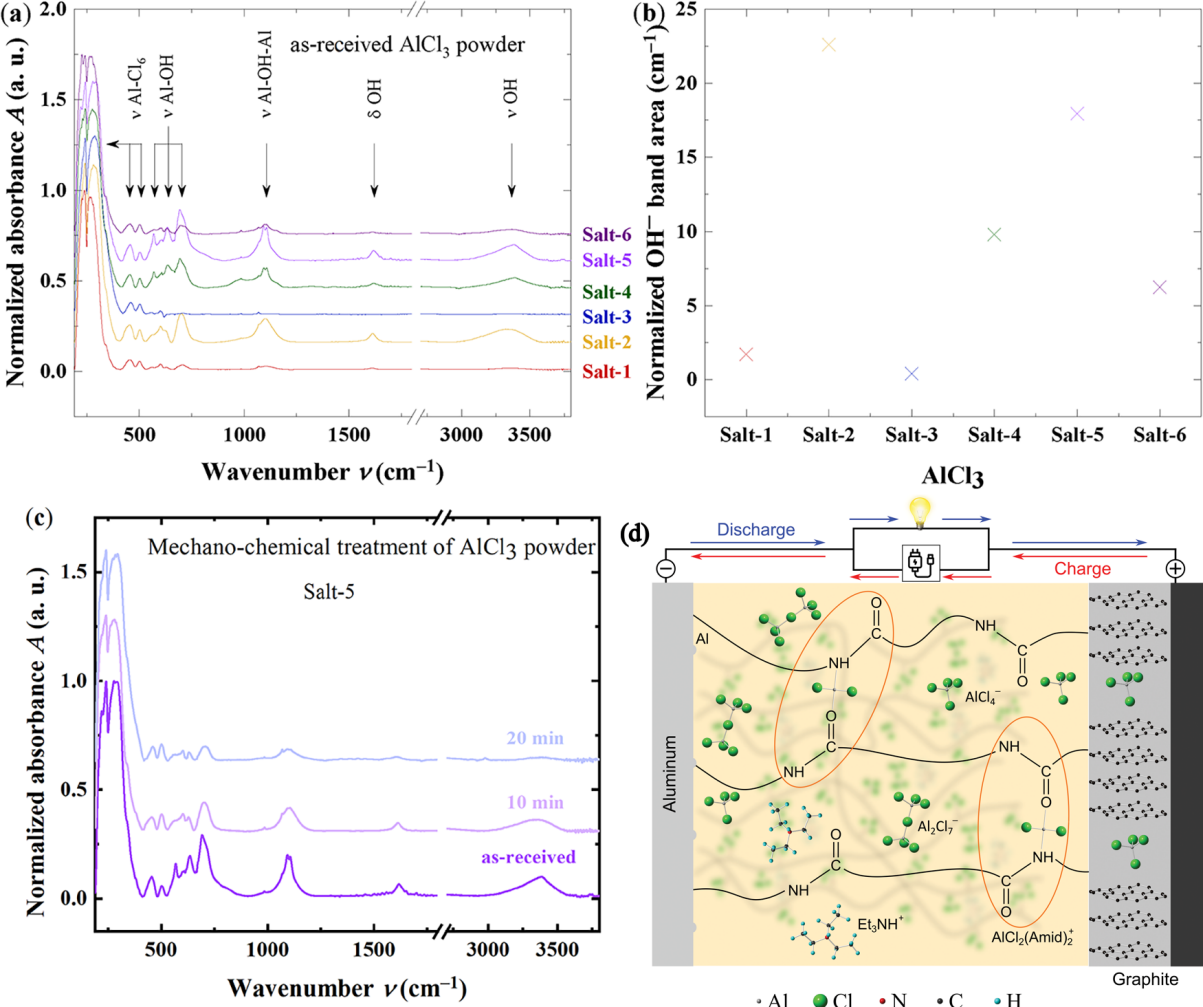
(a) ATR-FTIR spectra of six different AlCl_3_ powders with varying purities and moisture levels, (b) integrated area of the O–H stretching band, proportional to moisture content, (c) grinding of granular AlCl_3_ into fine powder as a function of time, illustrating mechanochemical treatment and associated out-diffusion of moisture, and (d) schematic of the polymer complex and ion transport in an Al-polymer battery. Polymer chains coordinate with the [AlCl_2_(Amide)_2_]^+^ cations. Gray, green, red, dark gray, and cyan circles represent Al, Cl, N, C, and H atoms, respectively. The diagram illustrates Al as the anode, graphite as the cathode, and a PA-based SPE as the electrolyte. During charging/discharging, AlCl_4_^–^ ions reversibly intercalate/deintercalate into/from graphite, while Al undergoes deposition/dissolution at the anode.

To quantify the moisture level of each AlCl_3_ salt, the integrated O–H band areas were calculated and are presented in Figure 1b. These integrated areas are proportional to the water content. The band intensities of the O–H species vary by more than a factor of 56 across the different salts. Among the samples, salt-2 exhibits the highest moisture content, while salt-3 shows the lowest. The adsorbed water in the salts is weakly bound, and it is well-known that hydrogen-containing species can diffuse out as a result of mechanochemical treatment, such as grinding under reducing conditions^40^ (in this case, an argon-filled glovebox). This effect is illustrated in Figure 1c, where sample salt-5 was ground into a fine powder. As a function of grinding time, the water content decreases significantly, and after 20 min, the spectrum closely resembles those of salt-6 or salt-1. During sample preparation (ILs and SPEs), the granular salts (salt-4 to salt-6) were ground for 5 to 15 min prior to use.

Manufacturer specifications (see Table S3) report water contents of <2000 ppm for salt-2 and <100 ppm for salt-1 and salt-5, which only partially align with our findings. The deeper yellow coloration of the ionic liquids formed with salt-2 and salt-5 (Figure S3A) further supports their higher moisture content, whereas the lighter color of salt-1 corresponds well with the observed lower water content. The clear, transparent solution formed by salt-4 and 6 may result from mechanical effects during grinding.

### Electrochemical Functionality of SPE AIBs

The IL system composed of AlCl_3_ and Et_3_NHCl, along with its operation in AIBs, has been detailed by Xu et al.^41^ The working principle of the IL-based SPE closely resembles that of a conventional IL system. When a polymer is incorporated into the IL system (AlCl_3_/Et_3_NHCl = 2:1) at a 1:1 molar ratio of IL to polymer, the resulting polymer electrolyte primarily contains AlCl_4_^–^ species (as shown in the reaction below), making the system neutral–similar to an IL with a 1:1 AlCl_3_/Et_3_NHCl ratio. However, upon the addition of “excess AlCl_3_” (typically in the range of 0.1 to 1 mol, resulting in a total of 2.1 to 3 mol AlCl_3_ in the polymer system), Al_2_Cl_7_^–^ ions are regenerated, thereby shifting the system toward Lewis-acidic composition.^10^ This acidic environment enables reversible Al deposition and dissolution, thereby maintaining battery operation. Additionally, the cationic complex [AlCl_2_(Amide)_2_]^+^ may form, acting as a network binder within the polymer matrix and facilitating ion transport.^10^ During charging, AlCl_4_^–^ intercalates into the graphite cathode, while Al is deposited at the anode via reduction of Al_2_Cl_7_^–^.^42^ These reactions reverse during discharge: Al is oxidized at the anode, and AlCl_4_^–^ deintercalates from the cathode. A schematic illustration of the ionic polymer complex system, including the ion transport mechanism and charge–discharge processes, is presented in Figure 1d. It also depicts the coordination of [AlCl_2_(Amide)_2_]^+^ with carbonyl (C=O) groups on adjacent polymer chains, as described by Mohammad et al.^10^ The associated electrochemical reactions are as follows

Ionic liquid formation

1 $${\mathrm{Et}}_{3}\mathrm{NHCl}+{\mathrm{AlCl}}_{3}↔{\mathrm{Et}}_{3}{\mathrm{NH}}^{+}+{\mathrm{AlCl}}_{4}^{-}\;\left(r=1\right)\;\left(\mathrm{neutral}\right)$$

2 $${\mathrm{Et}}_{3}\mathrm{NHCl}+2{\mathrm{AlCl}}_{3}↔{\mathrm{Et}}_{3}{\mathrm{NH}}^{+}+{\mathrm{Al}}_{2}{\mathrm{Cl}}_{7}^{-}\;\left(r=2\right)\;\left(\mathrm{acidic}\right)$$

where *r* represents the molar ratio of AlCl_3_ to Et_3_NHCl. In our study, an IL system with a molar ratio of *r* = 2 was used. Upon the addition of 1 mol of polymer to this IL system, the following reactions occur

Polymer complex

3 $${\mathrm{Al}}_{2}{\mathrm{Cl}}_{7}^{-}↔{\mathrm{AlCl}}_{4}^{-}+{\mathrm{AlCl}}_{3}$$

4 $$2{\mathrm{AlCl}}_{3}+2\mathrm{Amide}↔{\mathrm{AlCl}}_{4}^{-}+{\left[{\mathrm{AlCl}}_{2}{\left(\mathrm{Amide}\right)}_{2}\right]}^{+}\;\left(\mathrm{without}\;\mathrm{extra}\;{\mathrm{AlCl}}_{3},\;\mathrm{neutral}\right)$$

When an additional 0.5 mol of AlCl_3_ was introduced during polymerization, the system underwent the following reaction

5 $${\mathrm{AlCl}}_{4}^{-}+{\mathrm{AlCl}}_{3}↔{\mathrm{Al}}_{2}{\mathrm{Cl}}_{7}^{-}\;\left(\mathrm{with}\;\mathrm{extra}\;{\mathrm{AlCl}}_{3},\;\mathrm{acidic}\right)$$

Full cell reactions

1. Cathodic

6 $$C_{n}\left[{\mathrm{AlCl}}_{4}\right]+e^{-}↔C_{n}+{\mathrm{AlCl}}_{4}^{-}$$

2. Anodic

7 $$\mathrm{Al}+7{\mathrm{AlCl}}_{4}^{-}↔4{\mathrm{Al}}_{2}{\mathrm{Cl}}_{7}^{-}+3e^{-}$$

### FTIR Analysis of SPEs

To investigate the vibrational characteristics of the SPE films prepared using six different purities of AlCl_3_ salts, ATR-FTIR measurements were performed. As shown in Figure 2, all spectra essentially exhibit the same band patterns in the range of (160–3800 cm^–1^), as reported by Mohammad et al.^10^ The spectral features fall into two main regions: chloraluminate species (AlCl_4_^–^, Al_2_Cl_7_^–^) and the PA matrix with the Et_3_NHCl additive.

**Figure 2:**
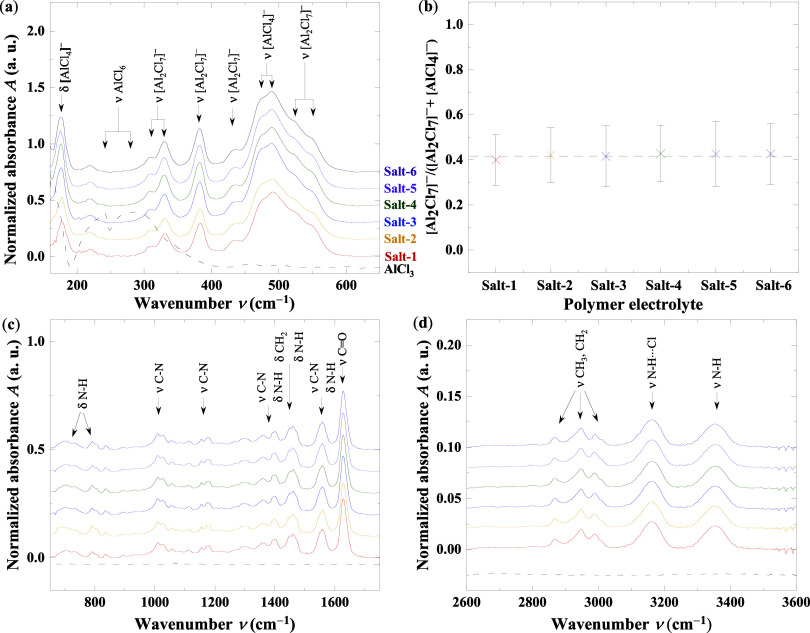
FTIR spectra of six SPEs prepared with different purities of AlCl_3_ salts, salt-1 to salt-6. ATR-FTIR spectra of various polymer electrolytes with a molar ratio of *r* = 1.25 for AlCl_3_/(PA6 + Et_3_NHCl): (a) chloroaluminate species (AlxCly) vibrational region, (b) band analysis of the Al_2_Cl_7_^–^ fraction in the polymers, (c) vibrational region of the polymer matrix and Et_3_NHCl, and (d) N–H and C–H stretching region. No water-related vibrational bands were observed, indicating that the water content is likely below the detection limit of the spectrometer.

Figure 2a highlights the vibrational modes of AlCl_4_^–^ and Al_2_Cl_7_^–^ species in the 160–650 cm^–1^ region, consistent with previous reports.^10,43^ Comparison with dry AlCl_3_ powder (salt-3, dashed line in Figure 2a) shows no overlap with the characteristic phonon modes of AlCl_6_ octahedra, indicating full dissociation and molecular dispersion of the salts within the polymer matrix. Figure 2b presents the pseudo-Voigt fitting used to quantify Al_2_Cl_7_^–^ content based on the integrated area ratio of the AlCl_4_^–^ and Al_2_Cl_7_^–^ stretching bands in the 450–600 cm^–1^ range for all salts electrolytes. A detailed description of the spectral deconvolution is provided in Supporting Section S.3.1, where Figure S5 presents the spectral fitting of the AlCl_4_^–^ and Al_2_Cl_7_^–^ modes for the polymer electrolyte based on Salt-1. However, in Figure 2b, all SPEs exhibit an average Al_2_Cl_7_^–^ content of (41.9 ± 0.4) %, consistent with previously reported values for the PA6-based system discussed above.^10^ These results suggest that neither the salt impurity nor the initial moisture content significantly affects the resulting Al_2_Cl_7_^–^/AlCl_4_^–^ equilibrium in the final SPE.

Figure 2c presents the mid-IR region, which is dominated by the vibrational modes of the PA polymer matrix and the Et_3_NHCl additive. These include characteristic vibrations associated with functional groups such as C–H and N–H bending as well as C–N and C=O stretching modes. Figure 2d focuses on the N–H and C–H stretching regions. All bond types, along with their vibrational modes and corresponding frequencies, are listed in Table S5 (see Supporting Information). Notably, no vibrational features indicative of residual water were detected in any of the SPEs, and no additional bands associated with impurity-related species were observed. This suggests that water was effectively removed during the annealing step and that the impurity concentrations were too low to produce detectable IR signatures.

### Electrochemical Kinetics and Aluminum Stripping/Plating Behavior

To evaluate the electrochemical kinetics and aluminum stripping–plating behavior of SPEs prepared using AlCl_3_ salts of varying purities, cyclic voltammetry (CV) measurements were performed on symmetric Al/SPE/Al cells. CV tests were performed on four batches of SPEs to ensure statistical reliability. A total of 24 coin cells were assembled using four batches of electrolyte, with each batch comprising six coin cells. This approach ensured the reliability of the results, verified by statistical analysis. The CV curves for batch-1 to batch-4 SPEs are presented in Figure 3a–d.

**Figure 3:**
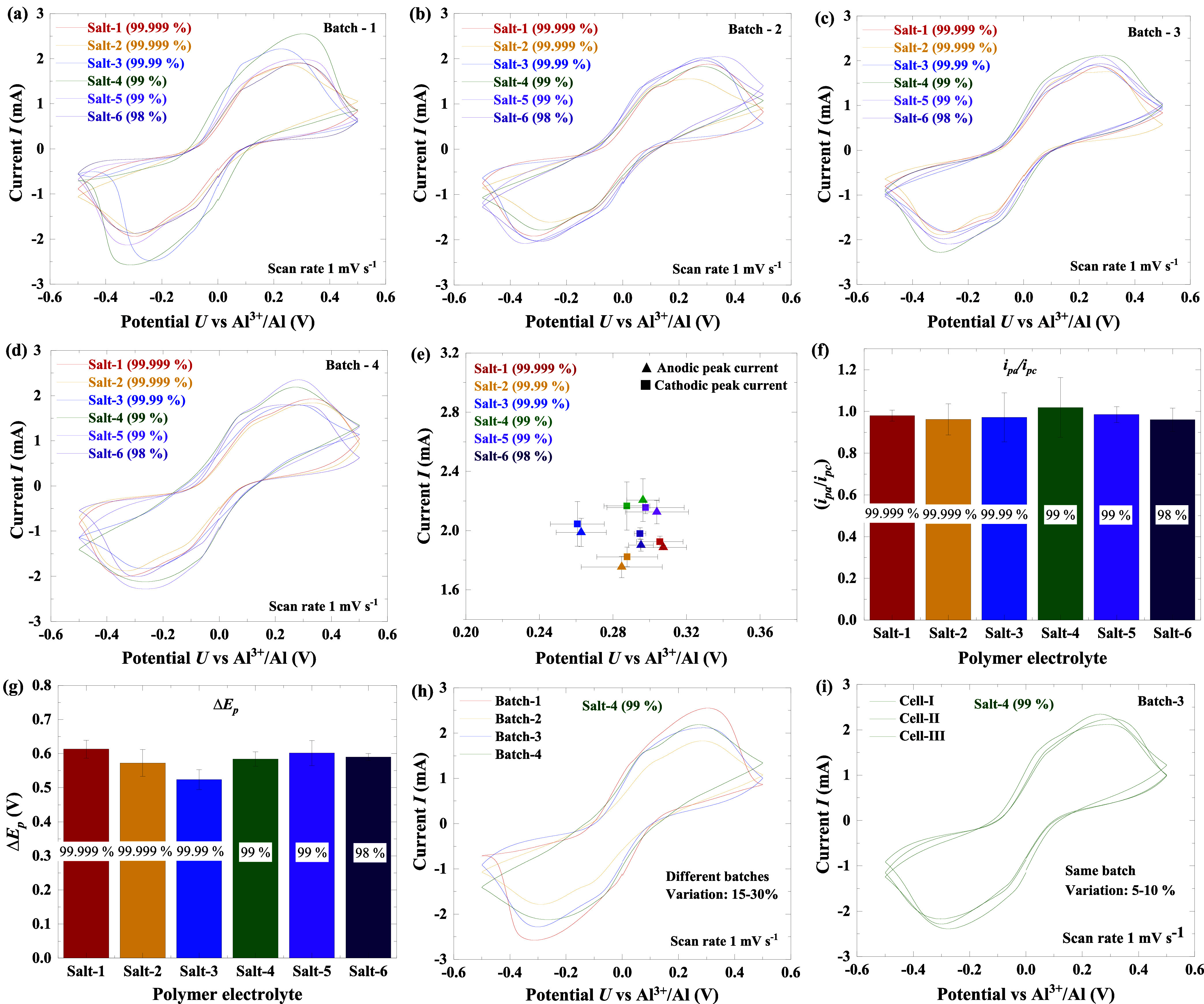
CV measurements: (a–d) CV curves of batch-1 to batch-4 SPEs, (e) average anodic and cathodic peak currents and their corresponding voltages derived from four batches of SPEs (24 samples) for each salt, (f) average peak currents ratios *i*_pa_/*i*_pc_, (g) average peak-to-peak separations Δ*E*_p_ of six salts SPEs derived from Figure e, (h) CV curves of salt-4 cells from four batches (batch-1 to batch-4), showing a significant variation in *i*_pa_ and *i*_pc_, attributed to the SPE preparation process, and (i) CV curves of three salt-4 cells from batch-3, showing a slight variation in *i*_pa_ and *i*_pc_, attributed to the coin cell assembly process.

From these figures, it is evident that all CV curves exhibit a characteristic duck-shaped profile with symmetrical anodic and cathodic branches. The voltammograms display well-defined anodic and cathodic peaks, corresponding to oxidation and reduction processes, respectively, indicating reversible redox behavior within the SPE system. The magnitude of these currents reflects the concentration of active species involved in the redox processes.^44^ However, slight variations in the stripping/plating curves, such as differences in shape, peak current, and peak potential, are observed both within the same batch and across different batches. These variations can be attributed to factors such as inconsistencies in the SPE preparation process, variations in coin cell assembly, and the presence of impurities.

Figure S6a shows the anodic and cathodic peak currents and their corresponding voltages extracted from 24 CV curves across different batches. Significant variability is observed within each batch, highlighting differences in SPE electrochemical performance. Peak currents range from 1.55 to 2.58 mA, and voltages from 0.22 to 0.35 V (see Supporting Table S6). These variations can be attributed to two main factors in addition to impurities: (1) the electrolyte preparation process, where slight differences in composition, mixing, and reaction uniformity affect performance; and (2) the coin cell assembly process, where variables such as electrolyte mass/thickness, electrode surface roughness, interfacial contact, and applied pressure may influence performance to a limited extent. A confrontation of the effects from electrolyte preparation and coin cell assembly processes is given later in this section.

Figure 3e displays the average anodic and cathodic peak currents and their corresponding voltages for each salt electrolyte, obtained from the batch data in Figure S6a. The average values of four samples for each salt were calculated with the associated uncertainties represented as ±1σ standard deviations. The peak currents and voltages exhibit noticeable differences in some cases without overlapping values. The cells utilizing salt-4 (99%) exhibit the highest peak currents, while those employing salt-2 (99.999%) show the lowest. Among the cells, salt-3 (99.99%) displays the lowest peak voltage, while the highest peak voltage is evident in the salt-1 cells (99.999%). Evidently, a pattern emerges where cells using electrolytes with lower salt purity (98% to 99%) exhibit larger peak currents compared to those with high-purity salts (99.999%). From this observation, the high currents observed for salt-4 are likely associated with the higher content of a benign impurity, Zn (31.10 ppm in salt-4), whereas the low peak currents for salt-2 may be attributed to the higher content of a detrimental impurity, S (53.86 ppm in salt- 2), as supported by compositional analysis (see Table 2).^19,20,24^ The correlation with overall cell performance will be studied in the following.

The *p*-values for the anodic and cathodic currents were evaluated by using one-way analysis of variance (ANOVA) across four batches of AlCl_3_ salts with different purities. The analysis, performed using Origin software, yielded *p*-values of 0.56 for the cathodic current (*i*_pc_) and 0.25 for the anodic current (*i*_pa_). In both cases, the *p*-values are greater than 0.05, indicating that although systematic trends are observed, the differences in electrochemical performance do not reach statistical significance under the tested conditions.

The electrochemical performance of the six salt-based electrolytes—including average anodic and cathodic peak currents, voltages, *i*_pa_/*i*_pc_ ratios, and peak-to-peak separation (Δ*E*_p_)—is summarized in Table S7. The average *i*_pa_/*i*_pc_ ratios (see Figure 3f) were close to unity for all salts, indicating highly reversible redox reactions in the SPEs, regardless of the AlCl_3_ purity. Figure 3g presents the average Δ*E*_p_ values, ranging from (523 ± 29) mV to (612 ± 29) mV, which reflect differences in reaction kinetics.^45^ A larger Δ*E*_p_ implies a higher energy barrier for electron transfer and thus slower electrochemical kinetics.^46^ Broad redox peaks further suggest sluggish charge-transfer kinetics during the electrochemical process. However, the relatively small variation in Δ*E*_p_ among samples points to only minor kinetic differences, consistent with variations in electrolyte viscosity and wetting at the electrolyte–cathode interface.^47,48^

The oxidation and reduction areas for 24 cells from different batches (Figure 3a–d) were integrated using EC-Battery Lab software. Figure S6b compares these areas with red and blue bars representing anodic and cathodic regions, respectively. Figure S6c shows the average integral areas with standard error (1σ), and Table S8 summarizes the data. High-purity salts (99.999%) like salt-1 and salt-2 exhibit lower integral areas, while lower-purity salts (98–99%) show higher values. Since the integrated CV area reflects the total charge transferred,^45^ the larger areas observed for the lower-purity salts may indicate that trace impurities, such as Fe and Zn in salt-4 and salt-5, enhance interfacial activity. Although reducible metal-ion impurities like Fe^3+^ and Zn^2+^ could, in principle, promote galvanic corrosion or pitting on aluminum, such effects are largely suppressed in the present SPE system. The polymer matrix limits impurity mobility, while the chloride-rich coordination environment stabilizes these species and suppresses their redox activity. At trace levels, these impurities may instead act as weak redox mediators through their Fe^3+^/Fe^2+^ and Zn^2+^/Zn couples, facilitating charge transfer and contributing to SEI stabilization. Secondary effects may also arise from transient coordination with the polymer, subtly modifying the local ionic environment.^11,19,20^ Nevertheless, one-way ANOVA performed across multiple batches yielded *p*-values greater than 0.05 for both anodic and cathodic currents, indicating that while such impurities may contribute to interfacial activity, their overall effect on electrochemical performance is not statistically significant under the tested conditions. A dominant or direct influence on the aluminum deposition morphology cannot be conclusively established in this study.

To evaluate variation in CV curves across and within electrolyte batches, measurements were performed by using salt-4. Figure 3h and i show CV curves from different batches and from batch-3 (Cells I–III), respectively. The curves from different batches display greater scattering than those from the same batch, indicating that both SPE and cell preparation contribute to the variability. Peak current variation was 15–30% between batches and 5–10% within a batch, suggesting that 10–20% of the variation arises from polymer preparation and 5–10% from cell assembly. Similar trends were observed for salt-3 from different batches and within batch-2 electrolytes (see Figure S6d,e).

Based on the CV analysis, it can be inferred that the performance of the SPE is primarily influenced by the preparation parameters of both the SPE and the coin cells, as well as by the presence of impurities in the AlCl_3_ salts. Certain effective species—such as Zn and Fe at moderate levels—may facilitate ionic activity, whereas detrimental impurities like S can degrade performance. However, based on the available data sets, accurately isolating and quantifying the individual effects of these impurities remains unfeasible.

### Determination of Specific Ionic Conductivity

The ionic conductivities of the polymer electrolytes were measured by using PEIS on Mo/SPE/Mo symmetric cells. A total of 24 cells were assembled from four SPE batches, with six cells per batch, to ensure a robust data set. Impedance data were analyzed, and the PEIS spectra were represented as Nyquist plots, as shown in Figures 4 and S7 (see Supporting Information). These figures reveal that each sample exhibits a characteristic depressed semicircle in the high-frequency region, followed by an inclined straight line in the low-frequency region. The semicircle observed in the high-frequency region is attributed to the Mo/SPE interface, while the inclined straight line in the low-frequency region corresponds to the diffusion-driven concentration gradient near the Mo/electrolyte interface.^49^

**Figure 4:**
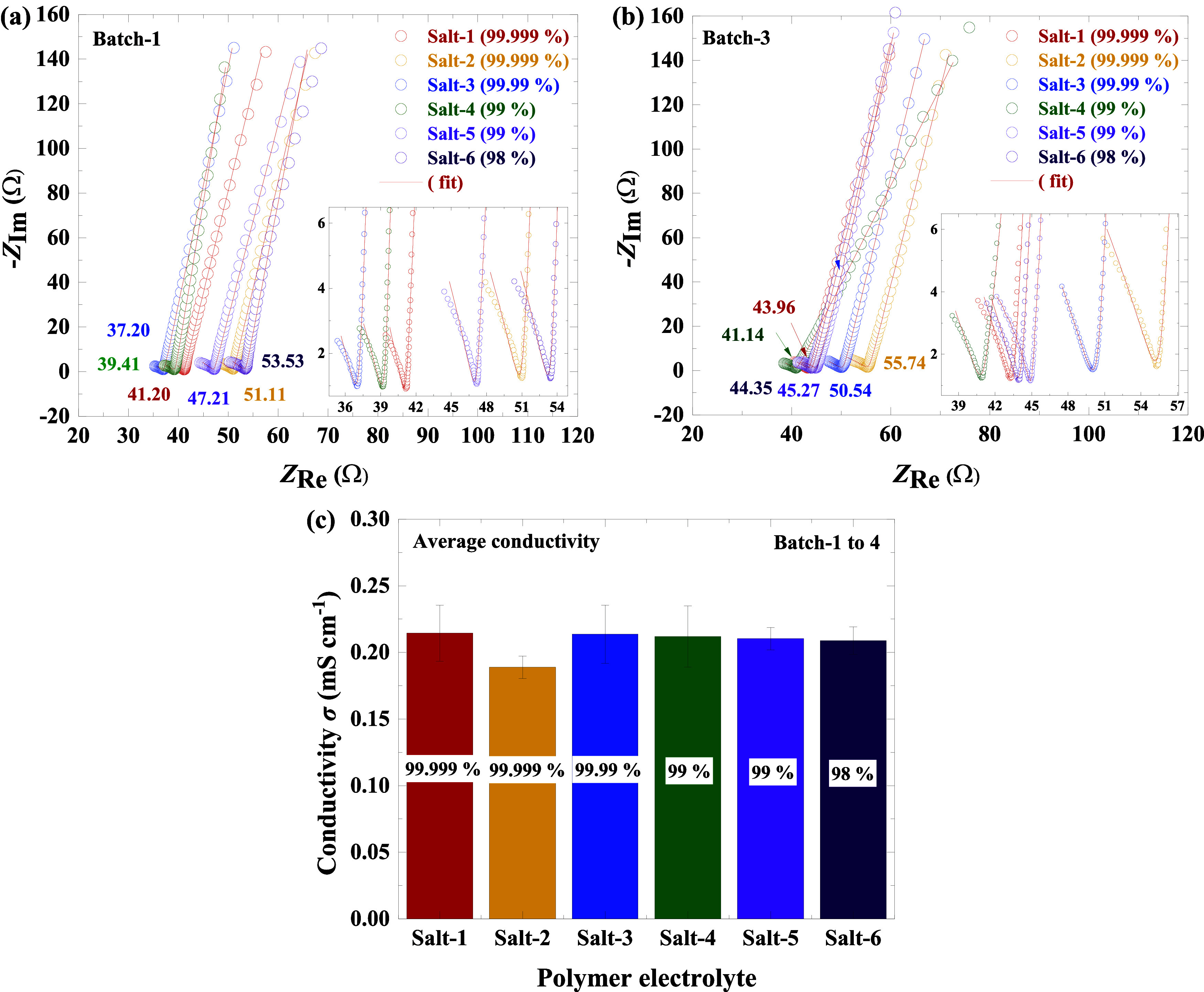
PEIS measurement curves: (a and b) represent batch-1 and batch-3 PEIS spectra with their fit and (c) average conductivities of salt-1 to salt-6 calculated from different batches (see Figure S7c), with 1σ standard error.

The intersection of the inclined line with the real impedance axis (*Z*_Re_) in the high-frequency region corresponds to the bulk resistance *R*_b_ of the electrolyte, which is used to calculate the conductivity of the samples. In an ideal ion-blocking electrode–interface system, the impedance spectra should exhibit a semicircle in the high-frequency region and a vertical line in the low-frequency region.^50^ However, in the current experiments, a depressed semicircle and an inclined straight line were observed instead. This deviation is likely due to factors such as electrode surface roughness and irregularities in the thickness and morphology of the polymer electrolyte.^51,52^ Figures 4 and S7 highlight the nonrepeatability of the PEIS spectra for a given salt sample, as the resistance varies between electrolyte samples from different batches. For quantitative analysis, the impedance spectra were curve-fitted using an equivalent circuit (see Figure S8), and *R*_b_ values were determined. The calculated conductivities for all 24 cells are presented in Figure S7c. The results show variations in conductivity across cells, likely due to differences in cell preparation parameters or inconsistencies at the electrode–electrolyte interfaces.

To further analyze the data, the average conductivity of each salt across four batches was calculated and is summarized in Table S9. A graphical representation of these average conductivities is shown in Figure 4c. All salt-based electrolytes, except salt-2, exhibit similar conductivities of around 0.21 mS/cm with slight variations. The conductivities obtained in this work are consistent with reported values for similar polymer electrolyte systems.^10^ In contrast, higher conductivities (1.1–5.77 mS cm^–1^) are typically observed in AIBs using IL-based electrolytes or separators, owing to enhanced ionic transport.^53,54^ The lowest conductivity observed for salt-2 is consistent with its higher sulfur concentration (Table 2), which likely contributes to performance degradation. Sulfur impurities may reduce conductivity by promoting the formation of insulating phases at electrode–electrolyte interfaces, altering the composition of the solid–electrolyte interphase (SEI), or deactivating electrochemically active sites, thereby hindering ion transport.^22–25^ Nevertheless, one-way ANOVA on four batches of AlCl_3_ salts with varying purities, performed using Origin software, yielded a *p*-value of 0.155, indicating that minor variations in the impurity content do not significantly affect the conductivity under the tested conditions. Taken together, both the statistical analysis and the averaged data confirm that these variations are minor and do not have a measurable impact on the overall battery performance.

### Ability of Electrolytes to Dissolve or Deposit Al, Stability Window, and Overpotential

The ability of the electrolytes to dissolve or deposit aluminum was evaluated using batch-3 electrolytes through chronoamperometry (CA) measurements on Al/SPE/Al symmetric cells at room temperature. The current–time curves, shown in Figure 5a, exhibit similar characteristic behavior with minor variations. Initially, the current drops sharply within the first minute and then gradually decreases until reaching a steady-state value. These features are indicative of underlying nucleation and growth processes occurring at the electrode surface.^55^ A broad peak–valley region is observed in the current–time curves for some measurements (e.g., salt-3 and salt-6 in Figure 5a), while other samples show only a smooth curvature. This variation in peak–valley characteristics may result from differences in electrode surface roughness and the local electrochemical environment.^56,57^ The current stabilizes at a nonzero value, reflecting a net current passing through the electrolyte system, driven by cationic movement^58^ and the continuous deposition of Al^3+^ ions originating from the cathode.^10^ The stabilized currents are similar across all electrolyte samples, with no significant changes observed in the CA curves or steady-state currents due to impurities. This suggests that impurities do not substantially impact the aluminum deposition or dissolution behavior in these systems.

**Figure 5:**
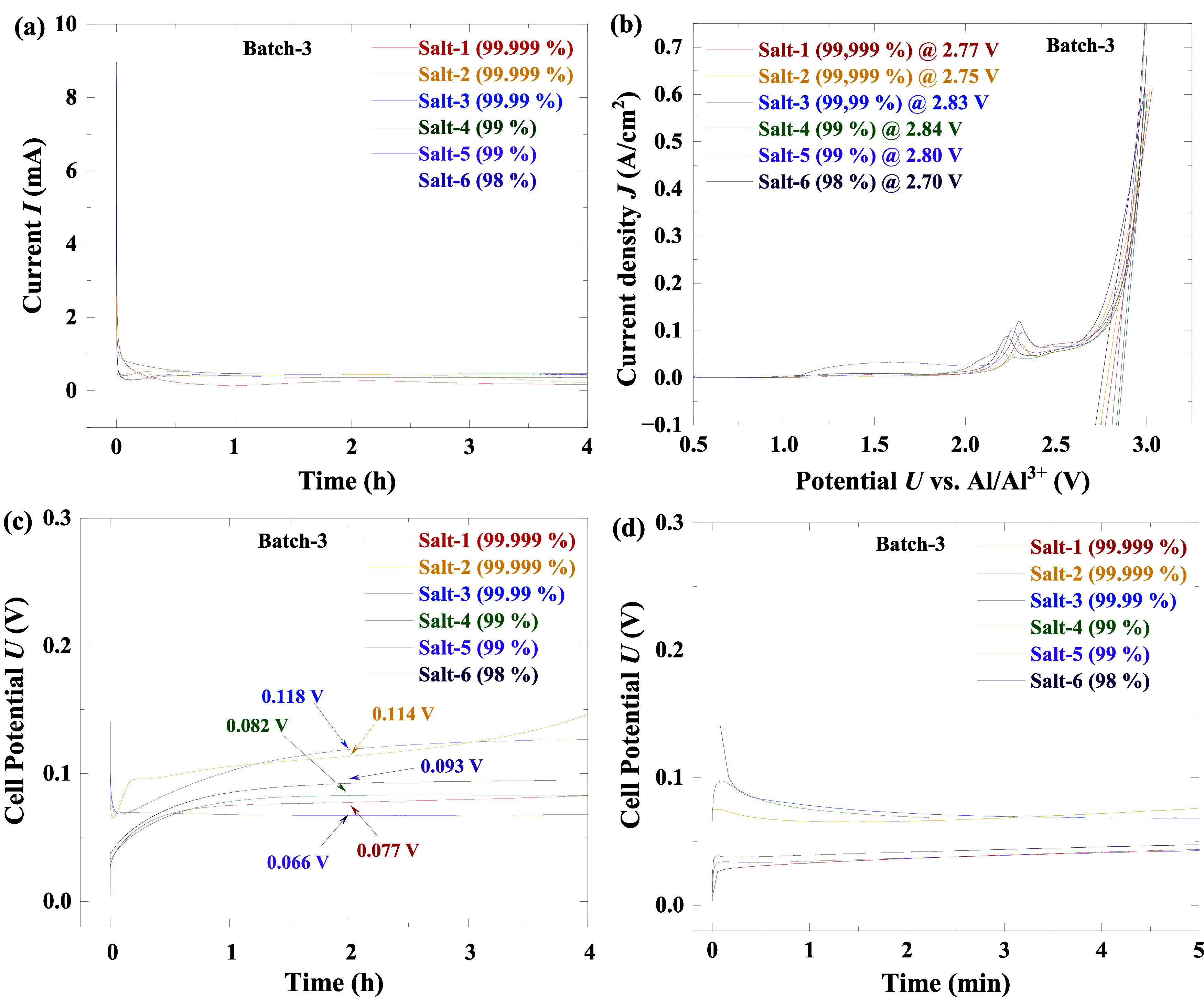
(a) CA measurement curves over 4 h of batch-3 SPEs, (b) LSV measurement curves with linear fitting of batch-3 SPEs, (c) chronopotentiometry measurement curves over 4 h, and (d) chronopotentiometry measurement curves over 5 min.

The stability windows of the SPEs were determined using linear sweep voltammetry (LSV) on Mo/SPE/Mo symmetrical cells, measured for batch-3 electrolytes (Figure 5b). Stability windows, obtained by linear fitting of the LSV curves, ranged from 2.70 to 2.84 V (Figures 5b and S9). All samples showed a gradual current increase followed by a sharp surge, attributed respectively to background Faradaic/non-Faradaic currents and oxidation reactions at the anode once the voltage exceeded the electrolyte’s stability window.^59^ These results align with reported windows of 2.6–2.8 V for both aqueous and nonaqueous Al battery systems.^9,60^ Notably, no significant differences in the stability window were observed across the tested samples, despite impurity levels ranging from 0.001–2%, suggesting that such impurities have minimal impact on the overall electrolyte stability. However, all LSV curves exhibited a small but distinct peak around 2.3 V, which may indicate interfacial processes such as Mo dissolution/deposition at the electrode surfaces,^61^ or parasitic reactions between the electrolyte and electrode materials that lead to gradual degradation before the onset of complete decomposition.

Chronopotentiometric measurements were conducted on batch-3 electrolytes using symmetric Al/Al cells at a current density of 200 μA/cm^2^ for 4 h (see Figure 5c). The overpotential, calculated as the midpoint between the initial and final voltages, ranged from 0.066 to 0.118 V. Among the samples, salt-2 and salt-3 exhibited the highest overpotentials, while salt-5 showed the lowest. As identical electrodes and procedures were used across all measurements, these differences are more likely attributed to subtle variations in electrodeposition/dissolution dynamics rather than to electrolyte impurities.^62^ Nonetheless, the overall differences in overpotential among the samples are relatively small. Additionally, an initial transient peak observed in most samples (see Figure 5d) likely corresponds to early electrochemical processes, such as surface activation and/or double-layer formation.^63,64^ These results indicate that the impurities present (0.001–2%) have minimal impact on such interfacial phenomena under the conditions studied.

### Electrochemical Kinetics of Full Cells and Determination of Specific Capacity, Efficiency, and Energy Density

The electrochemical behavior of the electrolyte system was further assessed in full cells. For this purpose, CV measurements were conducted on Al/SPE/SpG cells prepared using only batch-3 electrolytes, with the setup parameters detailed in the Electrochemical Testing section. The obtained cyclic voltammograms for the different salt electrolytes are illustrated in Figure 6a. All CV curves exhibit similar patterns, characterized by consistent shapes and well-defined redox peaks, confirming the reversible nature of the process and the stable energy storage properties of the electrolyte systems. While the redox peaks show minor variations, these changes do not follow a consistent pattern relative to the impurity levels. Additionally, no extra peaks associated with impurities were detected, suggesting that the impurities do not significantly impact the electrochemical behavior of the system.

**Figure 6:**
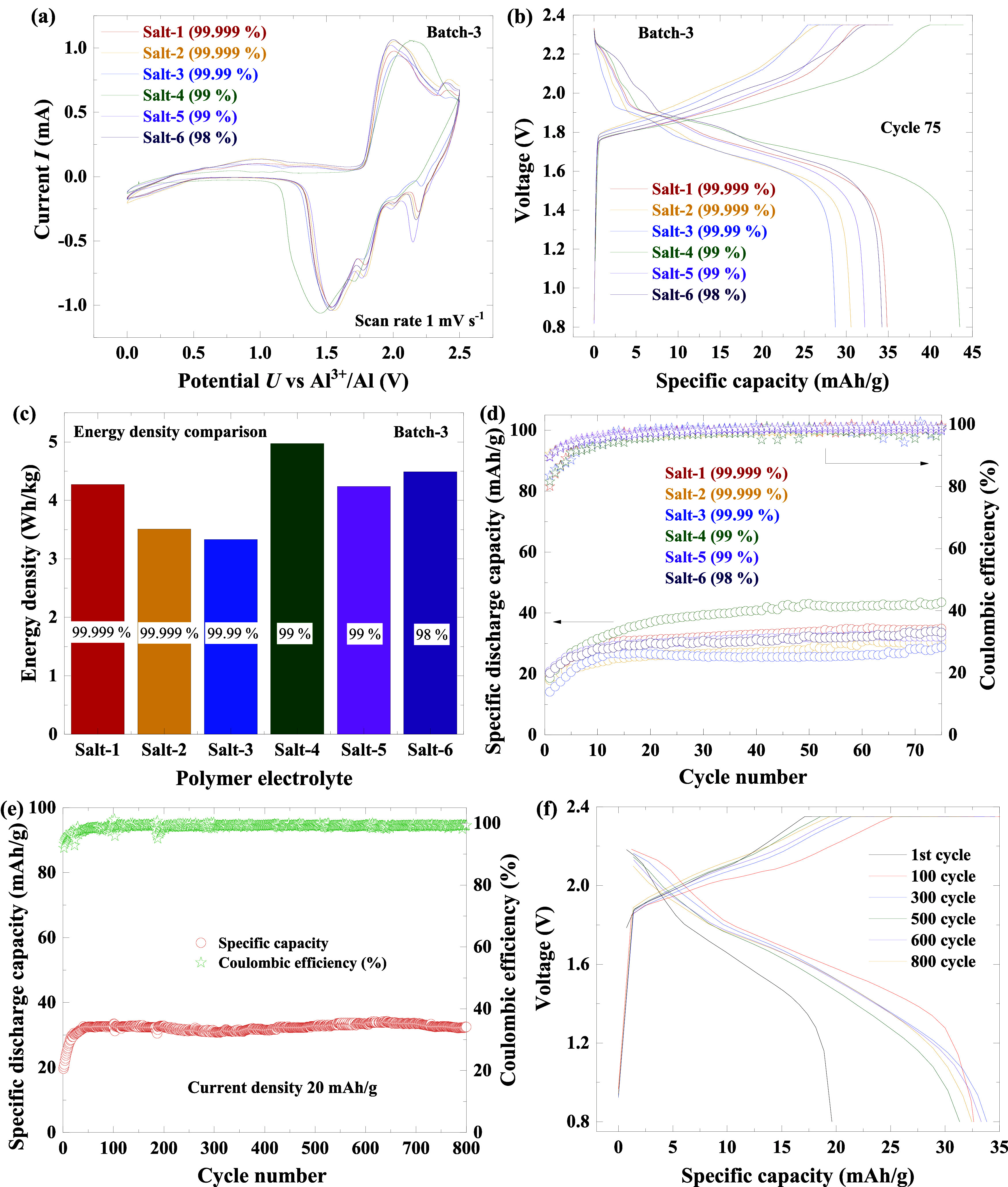
(a) CV curves of full cells (Al/SPE/SpG) from batch-3 electrolytes, (b) charge–discharge curves of the same set of cells at the 75th cycle, (c) energy densities, (d) specific discharge capacity and Coulombic efficiency versus cycle number up to 75 cycles, (e) specific discharge capacity and Coulombic efficiency versus cycle number for the salt-5 electrolyte up to 800 cycles, and (f) charge–discharge curves in the 1st, 100th, 300th, 500th, 600th and 800th cycle corresponding to Figure (e).

Specific capacities based on the mass of the cathode active material were evaluated over 75 cycles by using the same cells as those employed in the previous CV measurements. Figures 6b and S9b show the charge–discharge curves of all cells in the 75th and first cycles, respectively. The data reveal noticeable variations in capacity among the cells, with values ranging from approximately 28 to 43 mAh g^–1^ in the 75th cycle and from 14 to 21 mAh g^–1^ in the first cycle. The specific capacities obtained in this work are comparable with previously reported results for polymer electrolyte systems with similar material compositions.^10^ Higher capacities, in the range of ∼50–150 mAh g^–1^, have been reported for AIBs employing IL electrolytes or IL-based separator systems, mainly due to their higher ionic conductivity and faster ion transport.^53,54,65^ The comparatively lower capacity in the present SPE/graphite system may be attributed to limited wetting at the electrode–electrolyte interface and the high compaction density of the graphite cathode, both of which restrict ion accessibility.^10,66^ The highest capacity was observed with salt-4, whereas salt-3 exhibited the lowest. Although salt-4 contains higher Zn and Fe concentrations and salt-2 shows an elevated S content, performance does not correlate directly with any single impurity. Notably, the high capacity of salt-4 despite measurable sulfur and the lower capacity of sulfur-free salt-3 suggest that electrochemical behavior arises from the combined effects of multiple impurities, interfacial factors, and local electrolyte–cathode interactions. Further studies are required to fully elucidate these mechanisms.

Energy densities, calculated based on the masses of active materials (including both cathode and electrolyte), exhibited a trend similar to that of the capacities (see Figure 6c). The observed differences in capacity and energy density are likely related to both the previously discussed factors, including impurities and slight variations in cathode loading and homogeneity (see Supporting Table S10). Minor variations in cathode loading and microstructural uniformity are consistent with the observed capacity differences, as they limit active material utilization and hinder ion transport pathways.^67^ The influence of these various factors is evident in the absolute cell capacity data presented in Figure S10a (see the Supporting Information).

Figure 6d shows the specific discharge capacities and Coulombic efficiencies over 75 cycles for various electrolytes. Initially, specific discharge capacities increased linearly for all cells during the first 10 cycles, reflecting steady improvement. Beyond cycle 10, most cells exhibited a gradual rise in capacity up to 75 cycles with minimal fluctuations. This initial increase is likely due to improved cathode surface wetting, which enhances cathode–electrolyte interconnection during cycling.^10^ The gradual capacity growth may also stem from cathode modifications that improve accessibility for charge-carrying species, with repeated cycling potentially optimizing structural pathways and active sites for ion transport and storage. Coulombic efficiencies remained consistently high (97.9–99.6%) across all cycles (see Figure 6d), indicating fully reversible electrochemical reactions without side reactions. Notably, impurities in AlCl_3_ salts did not consistently affect specific discharge capacities, energy densities, or Coulombic efficiencies, and no adverse effects were observed over 75 cycles.

Long-term electrochemical stability of two cells was evaluated using 99% pure Salt-5 electrolyte over 800 and 200 cycles at 20 mA g^–1^ (see Figures 6e and S10c, respectively). Figure 6f presents the charge–discharge profiles of the cell, while the corresponding curves at selected cycles (1st, 100th, 300th, 500th, 600th, and 800th) are shown to illustrate the evolution of electrochemical behavior with prolonged cycling. Similar charge–discharge profiles for the 200-cycle test at selected cycles are shown in the Supporting Information (see Figure S10c,d). After an initial activation period, the capacity stabilized without observable decay, while the Coulombic efficiency remained consistently high at approximately 99%, indicating reversible and stable electrochemical behavior. Importantly, no progressive capacity fading or efficiency loss was observed within the tested cycling window, suggesting that impurity-related accumulation or slow degradation processes are effectively suppressed in the present SPE system. While extended cycling beyond 800 cycles may further elucidate ultralong-term effects, the results demonstrate that minor impurities in lower-purity AlCl_3_ do not induce measurable degradation under the investigated conditions.

For comparison, Mohammad et al.^53^ reported on the cycling stability and Coulombic efficiency (>99%) using salt-3 (99.99%) in a polyacrylonitrile (PAN)–imidazolium chloride (EIMCl)-based ionic liquid system coupled with a graphite electrode. This further underscores the versatility of polymer-IL systems and highlights that both high- and moderate-purity salts can deliver stable long-term performance under suitable conditions.

### Surface Morphology and Elemental Characterization of the Anode

A pouch cell was prepared using Salt-5 electrolyte to investigate the Al foil anode surface. The fabrication procedure of the pouch cell is briefly described in Supporting Section S.3.7. The cell was cycled at a current density of 20 mA g^–1^ for 20 cycles. After cycling, the cell was disassembled, and the anode morphology was characterized using photographic and SEM imaging. Representative photographic and SEM images are presented in Figures 7a–d and S11, respectively.

**Figure 7:**
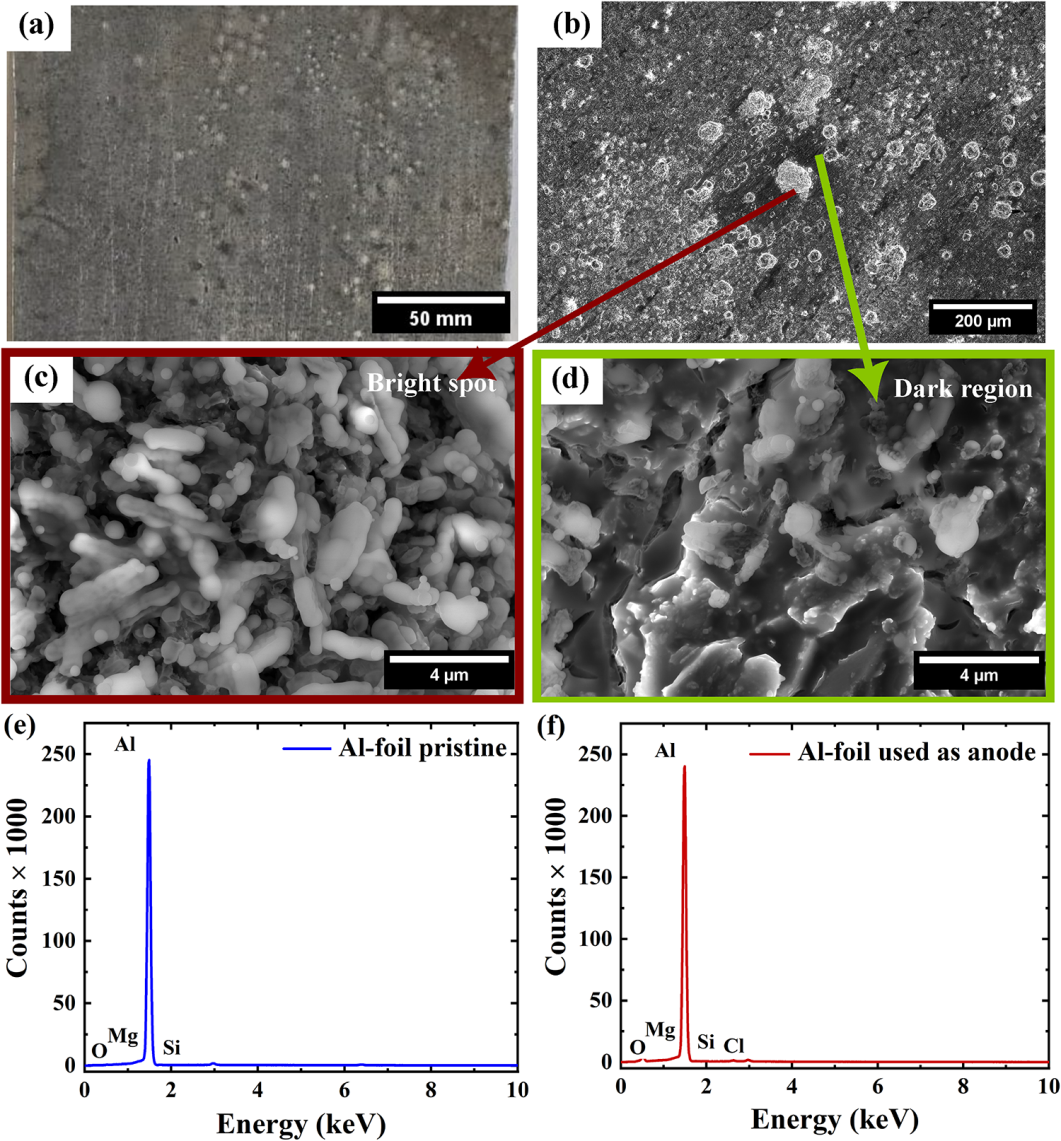
Photographic and SEM images of the Al foil anode from a pouch cell after 20 cycles at different magnifications: (a) Photographic image, (b) low-magnification SEM image, (c) higher-magnification SEM image showing a bright region, and (d) higher-magnification SEM image showing a dark region. EDX analysis of the Al foil: (e) EDX spectrum of pristine Al foil and (f) EDX spectrum of the Al foil used as the anode.

Figure 7a shows regions with both bright and dark contrasts on the Al foil surface. These contrasts are more clearly observed in the low-magnification SEM image (Figure 7b). The bright circular features are attributed to localized Al deposition, indicating that the deposition occurs as isolated particle growth rather than forming a continuous layer. In contrast, the darker regions correspond to areas with little or no Al deposition, likely associated with the absence of effective conductive pathways on the foil surface. High-magnification SEM images (Figure 7c,d) further resolve the fine structure of both regions, revealing clear morphological differences: The bright spots exhibit a finer, more granular structure compared to the relatively smooth or less-defined dark regions, distinguishing the deposited Al from the original substrate. Notably, no significant changes in deposition morphology attributable to impurity levels are observed, suggesting that impurities do not play a dominant role in governing the structural evolution under the investigated conditions.

The elemental composition of the pristine Al foil and the cycled Al anode was analyzed by EDX at multiple surface locations. Representative spectra from each sample are shown in Figure 7e,f, while Table S11 summarizes the detected elements and their relative contents. In both cases, Al is the predominant element, followed by O, indicating a native Al_2_O_3_ surface layer with trace amounts of Mg and Si. After cycling, a small amount of Cl is additionally detected at certain locations; however, its inconsistent presence suggests a localized and nonuniform distribution. The increased O content after cycling may be associated with enhanced surface roughness, leading to increased surface oxidation and a higher apparent oxygen signal. Overall, the similar elemental composition of pristine and cycled foils indicates that most detected species originate from inherent impurities in the Al foil, while the sporadic Cl signal is likely due to residual electrolyte species rather than systematic surface modification. No impurities associated with the electrolyte system or originating from AlCl_3_ were conclusively detected.

### Cost-Benefit Analysis

The price-to-performance ratio of polymer electrolytes prepared by using AlCl_3_ salts of varying purities was evaluated through comprehensive electrochemical characterization. Figure 8 illustrates the correlation between electrochemical performance and cost, with detailed data provided in Table S12 (see Supporting Information). The results indicate that the average performance of low-purity salts is comparable to or, in some cases, superior to that of higher-purity salts. For example, the cost of 99% purity AlCl_3_ (salt-5) is 0.027 Euros per gram, whereas ultradry (99.999%) AlCl_3_ (salt-1) costs 6.198 Euros per gram, which is ∼ 230 times higher (see Table 1). Despite this substantial price difference, the performance metrics (conductivity, capacity, Coulombic efficiency, and energy density) of the two salts do not differ significantly. Furthermore, salt-1 exhibited the highest conductivity and efficiency, salt-2 showed the lowest conductivity, and salt-4 had the lowest efficiency. Notably, salt-4 achieved the highest capacity and energy density, whereas salt-3 recorded the lowest capacity in both categories. These results demonstrate that peak performance is distributed across different salts, underscoring that higher-purity AlCl_3_ salts do not provide a significant performance advantage in polymer electrolytes.

**Figure 8:**
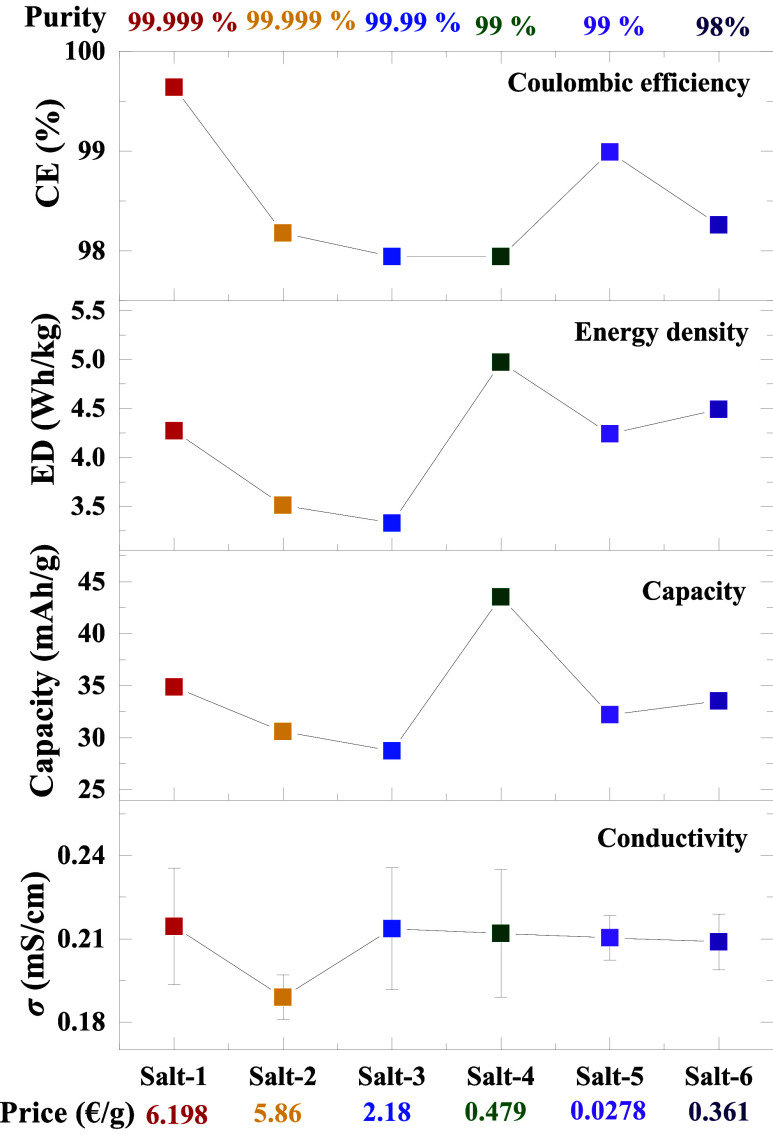
Performance of the different salt-based electrolyte samples and their costs per gram.

To further evaluate economic viability, the required amount of SPE for producing a CR2016 coin cell and the associated costs of AlCl_3_ salts at varying price points were estimated. This involved calculating the specific quantities of each material needed to formulate the electrolyte for a single cell. The estimated amounts per cell were approximately 0.0827 g of AlCl_3_, 0.0341 g of Et_3_NHCl, and 0.0280 g of polyamide (PA), resulting in a total electrolyte requirement of 0.145 g per cell. The cost of each electrolyte material and the relative cost of the SPEs fabricated with different purities of AlCl_3_ salts are summarized in Table 3. The cost of the other cell components used in our system was estimated at approximately 0.371 Euros (see Supporting Table S13), and this value was incorporated into the overall cell cost calculations shown in Table 3. The results reveal substantial variation in electrolyte costs depending on the purity grade of AlCl_3_. Specifically, using the lowest-cost AlCl_3_ salt reduces the SPE cost by approximately 94% and the overall cell cost by about 56% compared to the highest-cost salt. This notable cost reduction highlights the potential for significantly lowering Al-ion battery production expenses while maintaining the electrochemical performance.

**Table 3:** Estimated Cost of Electrolyte Materials Used per Cell Prepared Using Salt-1 to Salt-6, along with the Corresponding Total Cell Costs

salt AlCl_3_	purity (%)	AlCl_3_/cell (Euro)	Et_3_NHCl/cell (Euro)	PA/cell (Euro)	SPE cost/cell (Euro)	SPE cost reduction (%)	cell cost (Euro)	cell cost reduction (%)
1	99.999	0.513	0.005	0.025	0.543		0.914	
2	99.999	0.485	0.005	0.025	0.515	5	0.886	3
3	99.99	0.180	0.005	0.025	0.210	61	0.581	36
4	99	0.039	0.005	0.025	0.069	87	0.440	52
5	99	0.002	0.005	0.025	0.032	94	0.403	56
6	98	0.030	0.005	0.025	0.060	89	0.431	53

## Conclusion

This study systematically investigated the influence of inherent impurities in AlCl_3_ salts on the performance of PA–IL (AlCl_3_–Et_3_NHCl) SPEs for Al-ion batteries. AlCl_3_ salts with purities ranging from 98% to 99.999% and corresponding prices between 0.027 and 6.198 Euro/g were used to prepare ionic liquids (ILs), which were subsequently incorporated into SPEs. Comprehensive electrochemical characterization, including CV, CA, PEIS, LSV, and GCPL measurements, was performed on both symmetric and full cells. CV measurements conducted on symmetric cells revealed a highly reversible electrochemical process, with consistent Al stripping and plating behavior across all salt-based electrolytes. PEIS measurements yielded conductivity values ranging from (0.19 ± 0.01) to (0.21 ± 0.02) mS cm^–1^, with no significant trends related to impurities. CA tests revealed steady-state currents with typical exponential decay profiles showing no significant variations attributable to impurity levels. The electrochemical stability windows of the SPEs ranged from 2.70 to 2.83 V, unaffected by the impurity concentration.

CV measurements of full cells also demonstrated a comparable reversible nature, with specific discharge capacities ranging from 28.71 to 43.50 mAh g^–1^ and energy densities between 3.33 and 4.49 Wh kg^–1^. No systematic correlation between the electrochemical performance and impurity concentration was observed. All cells maintained high Coulombic efficiencies of 97.9% to 99.6% over 75 cycles, with negligible impact from impurities. Long-term stability and cycle life tests further confirmed that the electrochemical performance of the SPEs was unaffected by impurities, supporting the feasibility of using lower-purity AlCl_3_ salts for cost-effective Al-ion battery production. Complementary structural characterization, including SEM and EDX, revealed no significant impurity-induced differences on the Al anode surface, supporting the electrochemical findings.

In summary, the extensive electrochemical characterizations in this study revealed that impurities in commercial AlCl_3_ salts had a generally negligible impact on the electrochemical performance of SPEs for Al-ion batteries. However, the presence of beneficial impurities such as Fe and Zn may, to some extent, enhance electrochemical performance, whereas detrimental impurities such as S may lead to reduced conductivity and capacity. Impurities such as Na, Mg, and Ca remained passive and showed no observable impact on the electrochemical performance.

Cost analysis revealed a significant disparity: the high-purity salt (99.999% ultradry) was around 230 times more expensive than the low-purity salt (99%). By utilizing low-purity salt, the electrolyte cost can be reduced by approximately 94%, resulting in a ∼56% reduction in the overall battery cost. This demonstrates that employing low-purity AlCl_3_ salts presents a cost-effective strategy for large-scale Al-ion battery production, offering comparable electrochemical performance while substantially reducing production costs.

## Supplementary Material



## Notes

Additional Experimental Details and Results: electrolyte preparation, impurity characterization, cell components, FTIR spectra analysis, CV curve analysis, PEIS, charge–discharge test, specific discharge capacity and energy density calculation, SEM, and cost-benefit analysis (PDF)
