# Supplementary material for: A Cost Reduction Strategy for Aluminum–Polymer Batteries: The Role of Impurities within AlCl3
Source: ACS Omega. 2026 May 7;11(19):28159–74. doi: 10.1021/acsomega.5c13009 (PMC13191702; doi:10.1021/acsomega.5c13009)
Supplement: Supplementary file 1 [file ao5c13009_si_001.pdf]

Supporting Information

A cost reduction strategy for aluminum-ion batteries: The role of $AlCl_3$ impurities

Mohammad Mostafizar Rahman, Amir Mohammad, Shuvrodev Biswas, Thomas Köhler, Hartmut Stöcker, Dirk C. Meyer*

S.1. Introduction

A chronological evaluation of major aqueous and non-aqueous electrolytes for aluminum-ion batteries is provided in Figure S1.

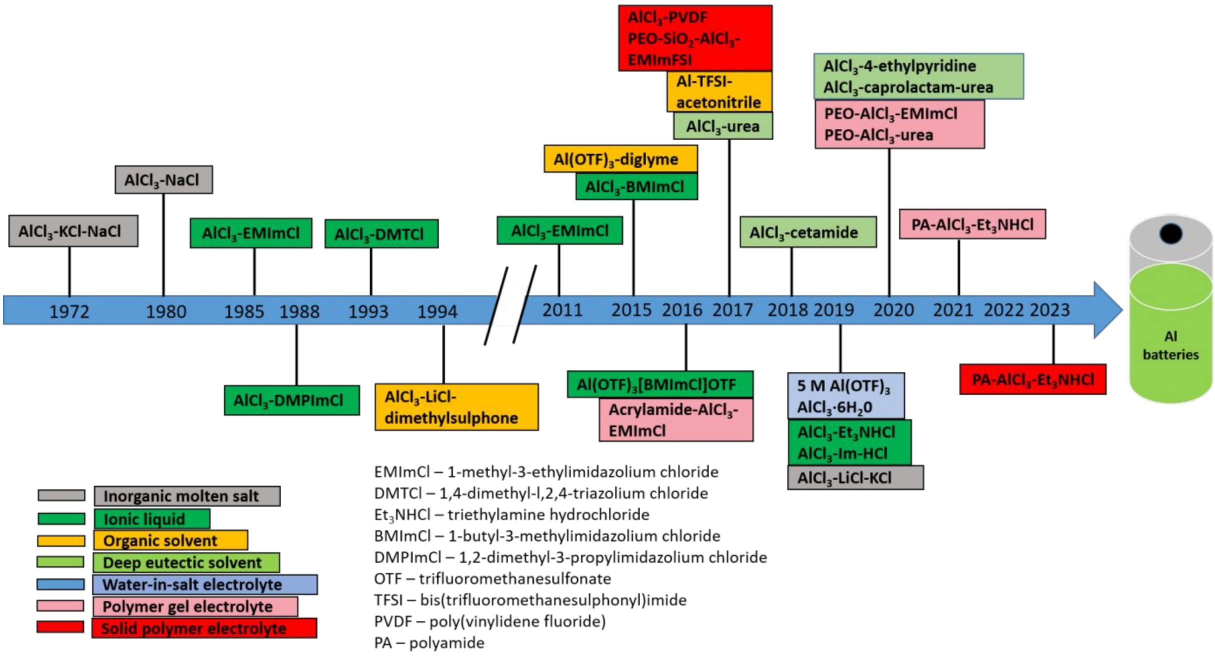

Figure S1. A timeline illustrating key aqueous and non-aqueous electrolytes used in Al batteries, adapted from Leung et al. [1]. Reproduced under terms of the CC-BY license [1], 2021, IOP Publishing Limited. The apparent gap between 1994 and 2011 reflects a period with very limited published work directly related to aqueous and non-aqueous electrolytes for aluminum batteries.

S.2. Experimental

S.2.1. Materials

The optical appearance of six $AlCl_3$ salts with varying purities, along with their respective purity levels and prices per 100 g, is displayed in Figure S2. Salts 1 to 5 were sourced from Alfa-Aesar, while salt-6 was obtained from Sigma-Aldrich.

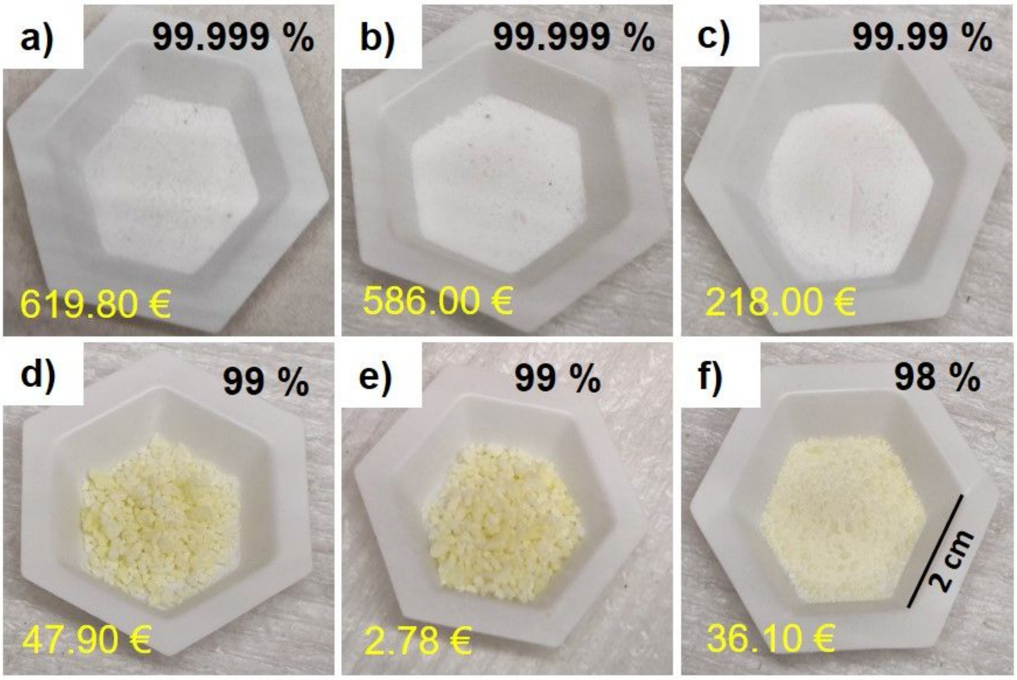

Figure S2. Optical appearance of six different purities of $AlCl_3$ salts (98–99.999%): Figures a to f correspond to salt-1 to salt-6. Salt-1 to salt-3 are white powders, while salt-4 to salt-6 are yellowish granules. The price per 100 g of $AlCl_3$ is provided for each salt.

S.2.2. Electrolyte preparation

Calculation of Extra $AlCl_3$ Amount:

The mass of additional $AlCl_3$ required for a target batch size was calculated using the following equation:

$$Mass_{\text{extra AlCl3}} = \frac{n_{\text{AlCl3}} \times M_{\text{AlCl3}} \times m_{\text{target SPE}}}{m_{\text{SPE}}} \quad \ldots\ldots\ldots\ldots(1)$$

where:

- $n_{\text{AlCl3}} =$ number of moles of extra $AlCl_3$ (e.g., 0.5 mol)
- $M_{\text{AlCl3}} =$ molar mass of $AlCl_3$ (133.33 g/mol)
- $m_{\text{target SPE}} =$ mass of the target SPE batch (e.g., 2 g)
- $m_{\text{SPE}} =$ mass of the total SPE formulation (e.g., 584.16 g)

Figure S3 shows salt-1 to salt-6 samples (ILs, mixture of ILs and PA, and SPEs) from batch-3 electrolytes. The ILs appeared as a clear and transparent solution, some with a yellowish colour, while the mixture of ILs and PA appeared milky white and viscous and the SPEs appeared as a

brown coloured solid polymer. The resulting polymer electrolytes exhibited enhanced mechanical properties compared to the starting materials and the intermediate mixture.

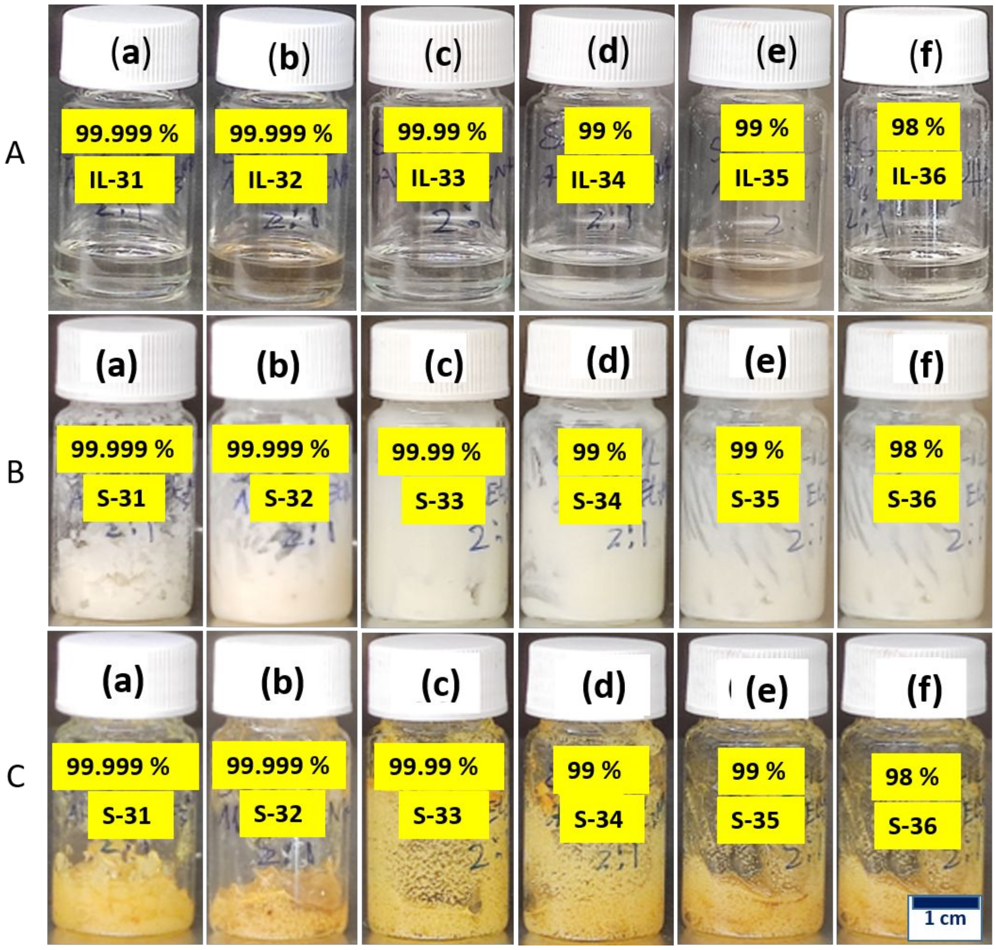

Figure S3. SPE preparation process with batch-3 electrolytes (salt-1 to salt-6): A) (a)–(f) ILs prepared using salt-1 (99.999%) to salt-6 (98%) after heating at 80 °C for 3 hours, B) (a)–(f) ILs and polymer (PA) mixture before heating and C) (a)–(f) Polymer electrolytes after annealing at 100 °C for 3 hours.

The four batches of ionic liquids (ILs) prepared using the six $AlCl_3$ salts are labelled in Table S1. The table includes the salt name, the ratio (r) of $AlCl_3$ to Et_3NHCl, and the purity levels of the respective salts.

Table S1. Identification of four batches of IL samples with their salt names, the ratio (*r*) of $AlCl_3$ to Et_3NHCl and the purity levels of the respective salts.

Sample	*r* of $AlCl_3/Et_3NHCl$	Batch-1	Batch-2	Batch-3	Batch-4	Purity of $AlCl_3$ (%)
Salt-1	2:1	IL-11	IL-21	IL-31	IL-41	99.999
Salt-2	2:1	IL-12	IL-22	IL-32	IL-42	99.999
Salt-3	2:1	IL-13	IL-23	IL-33	IL-43	99.99
Salt-4	2:1	IL-14	IL-24	IL-34	IL-44	99
Salt-5	2:1	IL-15	IL-25	IL-35	IL-45	99
Salt-6	2:1	IL-16	IL-26	IL-36	IL-46	98

The four batches of sold polymer electrolyte (SPEs) prepared using the six $AlCl_3$ salts are labelled in Table S2. The table includes the salt name, the ratio (*r*) of IL, $AlCl_3$ and PA, and the purity levels of the respective salts.

Table S2. Identification of four batches of SPE samples with their salt names, the ratio (*r*) of IL, $AlCl_3$ and PA, and the purity levels of the respective salts.

Sample	*r* of IL/$AlCl_3$/PA	Batch-1	Batch-2	Batch-3	Batch-4	Purity of $AlCl_3$ (%)
Salt-1	1:0.5:1	S-11	S-21	S-31	S-41	99.999
Salt-2	1:0.5:1	S-12	S-22	S-32	S-42	99.999
Salt-3	1:0.5:1	S-13	S-23	S-33	S-43	99.99
Salt-4	1:0.5:1	S-14	S-24	S-34	S-44	99
Salt-5	1:0.5:1	S-15	S-25	S-35	S-45	99
Salt-6	1:0.5:1	S-16	S-26	S-36	S-46	98

S.2.3. Impurity characterization

According to company data, the $AlCl_3$ salts contain various metals, including light, alkaline, transition, and some heavy metals, as well as moisture. However, our ICP-OES analysis detected only a limited number of elements in the salts. Table 2 (see main manuscript) presents our experimental results, while Table S3 summarizes the impurity contents provided by the manufacturers. Information regarding the impurity contents of salt-6 was not available from the company source.

Table S3. Impurities present in $AlCl_3$ salts in ppm according to manufacturers.

Element number	Impurity element	Salt-1 (ppm)	Salt-2 (ppm)	Salt-3 (ppm)	Salt-4 (ppm)	Salt-5 (ppm)	Salt-6 (ppm)
4	Be	–	–	–	<25	–	–
11	Na	0.2	0.3	–	–	–	–
12	Mg	2	2	20	–	–	–
14	Si	<2	2	1	–	–	–
19	K	<2	<0.5	–	–	–	–
20	Ca	0.5	0.3	5	–	–	–
22	Ti	<0.2	<0.2	–	–	–	–
23	V	–	–	–	<13	–	–
24	Cr	<0.2	<0.2	–	<25	<2	–
25	Mn	<0.1	<0.1	–	<25	–	–
26	Fe	<0.2	<0.2	0.4	<50	<20	–
27	Co			–	<50		–
28	Ni	<0.2	<0.2	–	<13	<4	–
29	Cu	<0.2	<0.2	–	<25	<2	–
30	Zn	<0.2	<0.2	–	<25	<6	–
33	As	–	–	–	<25	–	–
34	Se	–	–	–	<25	–	–
38	Sr	–	–	–	<50	–	–
42	Mo	–	–	–	<13	–	–
48	Cd	–	–	–	<7	–	–
50	Sn	–	–	–	<50	–	–
51	Sb	–	–	–	<25	–	–
56	Ba	–	–	–	<25	–	–
82	Pb	–	–	–	<25	<4	–
Total		<8	<6.4	26.4	<496	<38	–
Moisture		<100	<2000	–	–	<100	–

S.2.4. Cell construction

Table S4 presents the sequence of cell components – anode, solid polymer electrolyte (SPE), cathode, and spacer – for the different cell types, as well as the relative thicknesses employed in the construction of the cells. A schematic of an Al/SPE/Al cell, including all components is provided in Figure S4.

Table S4. Cell components and their thicknesses.

Cell type	Anode (µm)	SPE (µm)	Cathode (µm)	Spacer (µm)
Al/Al symmetric	Al foil (30)	200	Al foil (30)	Steel (500)
Mo/Mo symmetric	Mo foil (50)	200	Mo foil (50)	Steel (500)
Al/G full cell	Al foil (30)	200	Graphite (130)	Steel (500)

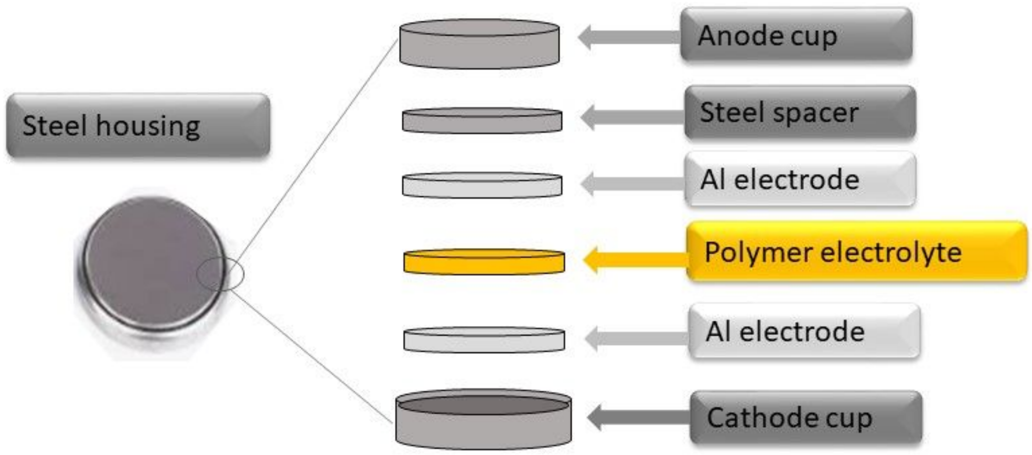

Figure S4. Schematic of an Al/SPE/Al symmetric cell.

S.3. Results and discussion

S.3.1. FTIR spectra analysis

The FTIR spectra of the PA6-based SPE electrolytes prepared with different $AlCl_3$ salts are shown in Figure 3 of the main manuscript, covering the frequency range of 160–3600 cm^{-1}. Table S5 summarizes the molecular vibrational modes observed in both the $AlCl_3$ salts and SPE films, identifying the associated functional groups along with their characteristic frequency ranges, including both stretching and bending vibrations.

Table S5. Molecular vibrations of $AlCl_3$ and PA6-based SPE films.

**Molecular vibrations of $AlCl_3$**		
**Frequency (cm^{-1})**	**Molecular group**	**Oscillation type**
3350	O-H	stretching
1615	O-H	bending
1090	Al-OH-Al	stretching
560-760	Al-OH	stretching
290-500	$AlCl_6$	stretching
**Molecular vibrations in ionic liquids of $AlCl_3$ and Et_3NHCl**		
**Frequency (cm^{-1})**	**Molecular group**	**Oscillation type**
3353	N-H	stretching
3164	N-H··Cl	stretching
2866–3015	CH_3, CH_2	stretching
1623	C=O	stretching
1557	N-H	bending
	C-N	stretching
1460	CH_2	bending
	N-H	bending
1360–1405	C-N	stretching
	N-H	bending
1008–1184	C-N	stretching
700–835	N-H	bending
550	$Al_2Cl_7^-$	stretching
525	$Al_2Cl_7^-$	stretching
491	$AlCl_4^-$	stretching
473	$AlCl_4^-$	stretching
432	$Al_2Cl_7^-$	stretching
381	$Al_2Cl_7^-$	stretching
329	$Al_2Cl_7^-$	stretching
307	$Al_2Cl_7^-$	stretching
175	$AlCl_4^-$	bending

Quantitative analysis of the polymer electrolyte:

For the quantitative analysis of the polymer electrolyte, the stretching modes of the $AlCl_4^-$ and $Al_2Cl_7^-$ species in the spectral range of 450 … 600 cm^{-1} were evaluated, following the approach reported by Mohamad et al. [2]. In this region, the two overlapping vibrational components of each species were selected and fitted, as the spectral shape in this range is most sensitive to the electrolyte composition [2]. The peak areas were determined using pseudo-VOIGT profiles, i.e., a linear combination of GAUSSian and LORENTZian functions [3]. The band area ratio was calculated as a/(a + b), where a and b correspond to the peak areas of $Al_2Cl_7^-$ and $AlCl_4^-$, respectively.

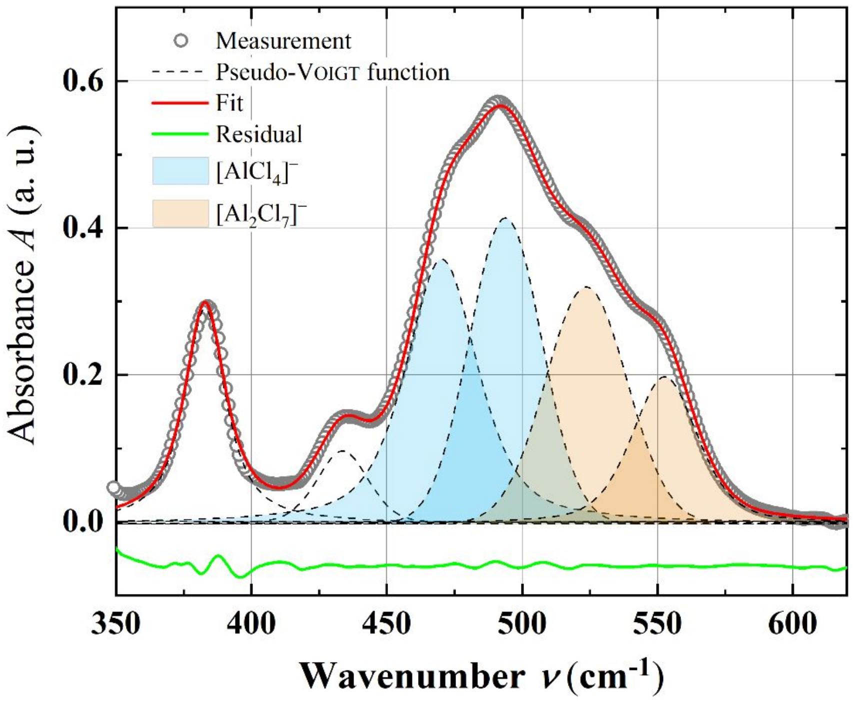

Figure S5. Spectral fitting of the $AlCl_4^-$ and $Al_2Cl_7^-$ modes for polymer electrolyte Sslt-1. Six pseudo-Voigt functions were used, with the two relevant modes of $AlCl_4^-$ and $Al_2Cl_7^-$ highlighted in blue and orange, respectively. The difference between the measured spectrum and the cumulative fit is shown with a vertical offset for clarity.

Figure S5 shows a representative spectrum of sample Salt-1. For all spectra, the same spline-based baseline correction was applied prior to fitting. Subsequently, the spectral region from 350 to 620 cm^{-1} was fitted using six pseudo-VOIGT functions. As shown in Fig. S5, the two vibrational modes of interest are highlighted in blue ($AlCl_4^-$) and orange ($Al_2Cl_7^-$). The corresponding peak areas were used to determine the band area ratio presented in Figure 2b of the main manuscript. In addition, Figure S5 shows the small difference between the cumulative fit and the measured spectrum, which is vertically offset for clarity.

S.3.2. CV curve analysis

Figure S6a shows the anodic and cathodic peak currents and corresponding voltages of the 24 samples from four batches of electrolyte. Figure S6b presents the integral areas of the CV curves of 24 samples from four batches and S6c represents the average integral areas of the anodic and cathodic parts. Figure S6e and f demonstrate the differences in the CV curves observed between different batches and within the same batch of batch-2 SPEs.

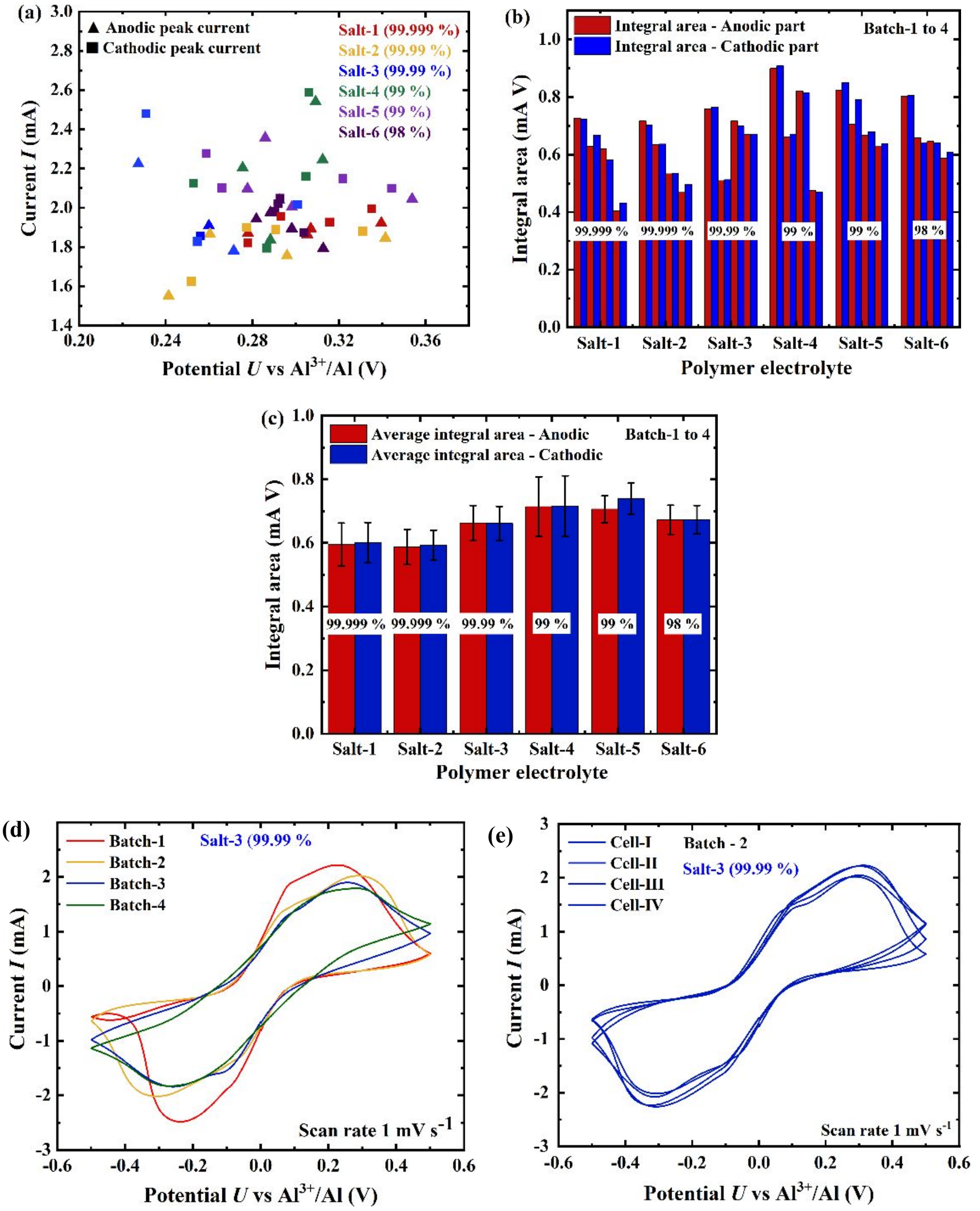

Figure S6. CV measurements: a) anodic and cathodic peak currents and their corresponding peak potentials of 24 electrolyte samples from four batches, b) integral areas of anodic and cathodic parts of four batches of electrolyte, c) average integral areas of anodic and cathodic parts with 1σ standard error, d) CV curves of salt-3 cells from four batches (batch-1 to batch-4) and e) CV curves of four salt-3 cells from batch-2.

Table S6 presents the peak current and potential ranges for different batches of electrolyte, while Table S7 summarizes key electrochemical properties derived from CV curves. Additionally, Table S8 reports the average integral areas of the anodic and cathodic regions.

Table S6. Anodic and cathodic peak current and voltage ranges of different batches.

Batch	Anodic peak current range (mA)	Anodic peak voltage range (mV)	Cathodic peak current range (mA)	Cathodic peak voltage range (mV)
Batch-1	1.86–2.53	227–309	1.87–2.58	230–321
Batch-2	1.55–2.04	241–353	1.62–2.09	251–344
Batch-3	1.75–2.24	259–312	1.82–2.15	256–304
Batch-4	1.78–2.35	271–341	1.82–2.27	252–335

Table S7. Anodic and cathodic peak currents and their corresponding peak voltages averaged over four batches as well as i_{ap}/i_{cp} and ΔE_p with their individual standard errors.

Salt	i_{pa} (mA)	E_{pa} (mV)	i_{pc} (mA)	E_{pc} (mV)	i_{pa}/i_{pc}	ΔE_p (mV)
Salt-1	1.88±0.013	307±13	1.92±0.037	−305±13	0.980±0.026	612±26
Salt-2	1.75±0.072	284±22	1.82±0.066	−287±17	0.962±0.075	572±39
Salt-3	1.98±0.095	262±14	2.04±0.150	−260±15	0.972±0.118	523±29
Salt-4	2.20±0.144	296±9	2.16±0.162	−287±12	1.018±0.143	584±21
Salt-5	1.90±0.039	303±7	1.97±0.038	−297±20	0.985±0.038	602±37
Salt-6	2.12±0.079	295±7	2.15±0.042	−294±3	0.960±0.056	590±10

Table S8. Averaged anodic and cathodic integral areas with standard error of the mean across four batches, as depicted in Figure S5c).

Salt	Average anodic integral area (mA V)	Average cathodic integral area (mA V)
Salt-1	0.59±0.068	0.60±0.063
Salt-2	0.59±0.055	0.59±0.047
Salt-3	0.66±0.055	0.66±0.054
Salt-4	0.71±0.094	0.72±0.095
Salt-5	0.71±0.042	0.74±0.049
Salt-6	0.67±0.042	0.67±0.045

S.3.3. Determination of specific ionic conductivity

The PEIS spectra of four batches of electrolyte along with their corresponding curve fitting are presented in Figure 4 (see main manuscript) and Figure S7. Specifically, Figures 4a and b display the PEIS spectra for batch-1 and batch-3, while Figures S7a and b shows the PEIS spectra for batch-2 and batch-4 electrolyte samples.

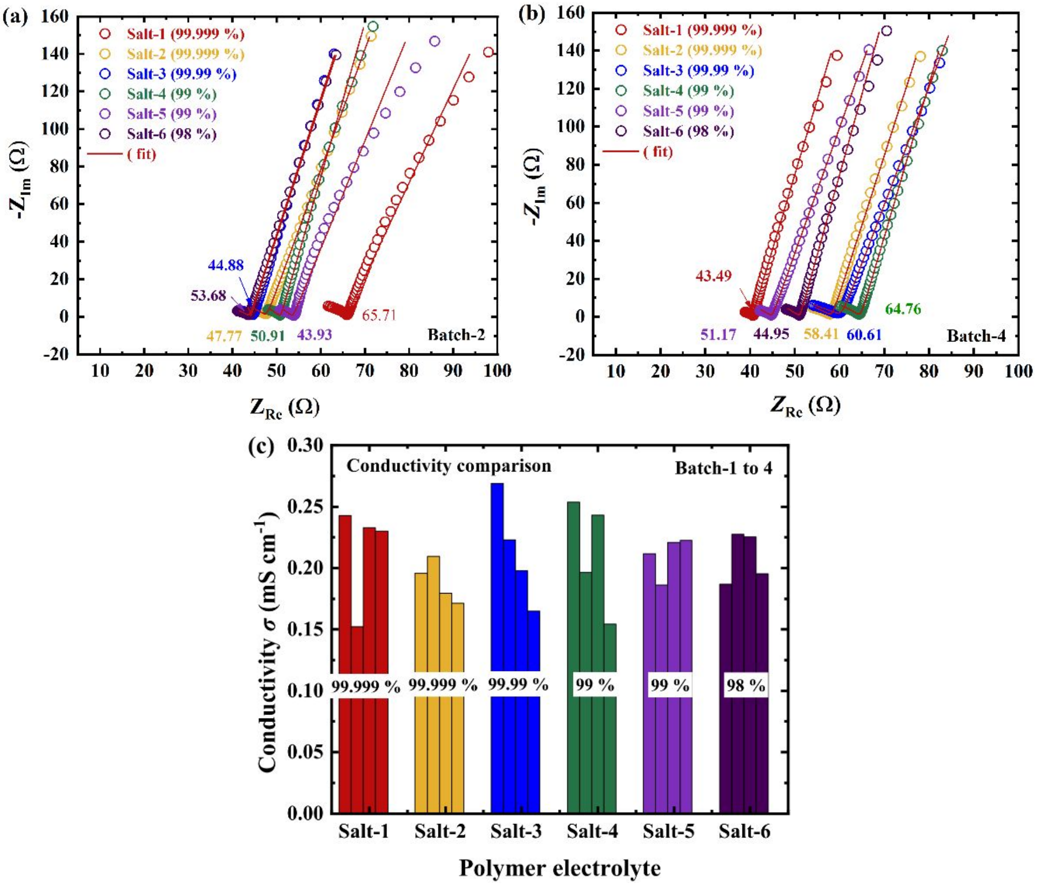

Figure S7. PEIS measurements: a) and b) represent batch-2 and batch-4 electrolytes and c) conductivities of 24 electrolyte samples derived from four batches of PEIS spectra from batch-1 to batch-4.

The equivalent circuit in Figure S8 was used to fit all PEIS curves, where R_b and C_g denote the bulk resistance and the geometry capacity of the SPE, respectively, CPE_1 represents the inhomogeneous electrolyte-electrode interface and CPE_2 represents the effects of dipolar relaxation in the electrolyte. The high-frequency semicircle stems from the combination of R_b and C_g and CPE_2, while the straight line after the semicircle can be explained with CPE_1 [2].

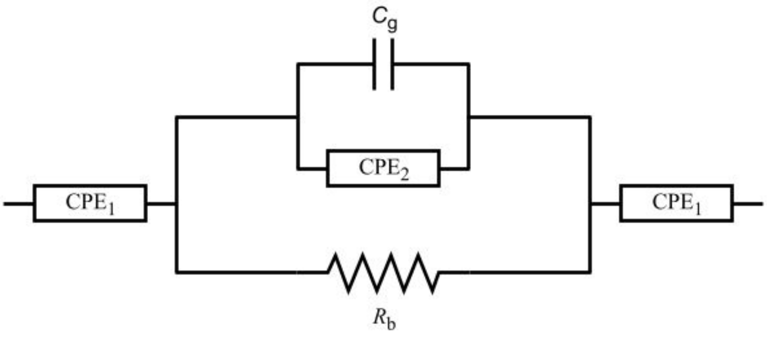

Figure S8. Equivalent circuit used to fit all PEIS spectra.

The conductivity of the polymer electrolytes σ was calculated using the equation:

$$\sigma = \frac{l}{R_b A} \text{ ....(2)}$$

where l is the thickness of the SPE, A is the area of the electrode contacting with the SPE and R_b is the bulk resistance. The expected values for the thickness of the SPE and the surface area of the electrode were used to determine the conductivity for each electrolyte sample. The calculated conductivities of 24 cells are presented in Figure S6c and the average conductivities of each salt electrolyte derived from Figure S6c are listed in Table S9.

Table S9. Polymer electrolyte sample thicknesses, surface area of electrodes and average conductivities of batch 1 to batch 4 electrolytes with their standard errors at 1σ confidence.

Name of sample	SPE thickness l (mm)	Electrode surface area A (cm^2)	Av. conductivity σ (mS/cm)
Salt-1	0.2	2.0	0.214±0.021
Salt-2	0.2	2.0	0.188±0.008
Salt-3	0.2	2.0	0.213±0.022
Salt-4	0.2	2.0	0.211±0.023
Salt-5	0.2	2.0	0.208±0.008
Salt-6	0.2	2.0	0.210±0.010

S.3.4. Determination of stability window

The LSV measurements were taken using a BioLogic SP300 potentiostat at a scan rate of 1 mV s^{-1} in the potential range of 0.5 V to 3 V. The stability windows determined for batch-3 electrolytes are shown in Figure S9.

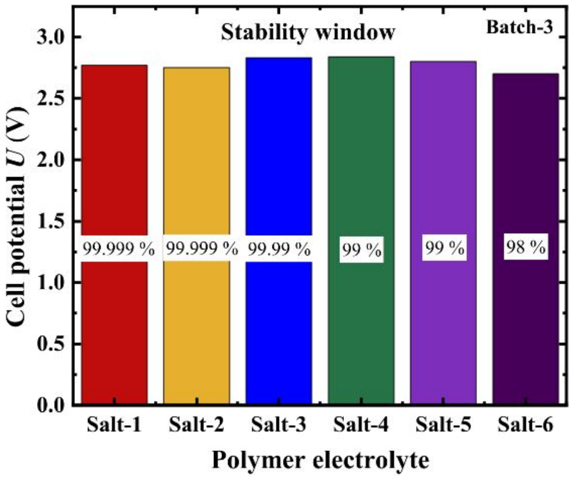

Polymer electrolyte

Figure S9. LSV measurement curves with linear fitting of batch-3 electrolyte samples. Comparison of stability windows of batch-3 electrolyte samples.

S.3.5. Charge-discharge test

The specific charge-discharge capacity curves of 1st cycle are shown in Figure S10a. The cell capacity reflects the actual charge storage capability of the cell, accounting for differences in cathode loading, inhomogeneity and material utilization. The absolute cell capacities of polymer electrolytes prepared with six different $AlCl_3$ purities are presented in Figure S10b. Long-term cycling of the third cell prepared with Salt-5 electrolyte, up to 200 cycles, is shown in Figure S10c, while the corresponding charge–discharge profiles, including the initial cycles, are presented in Figure S10d.

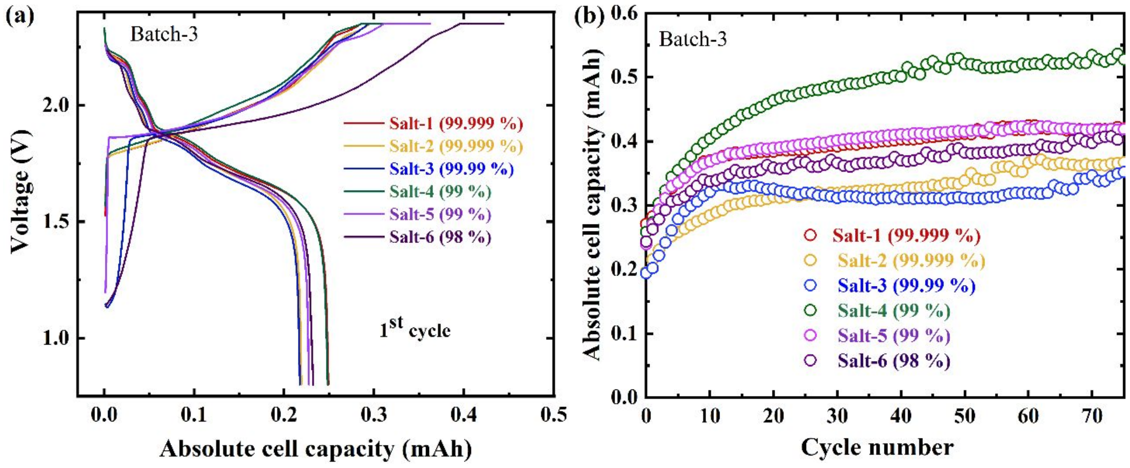

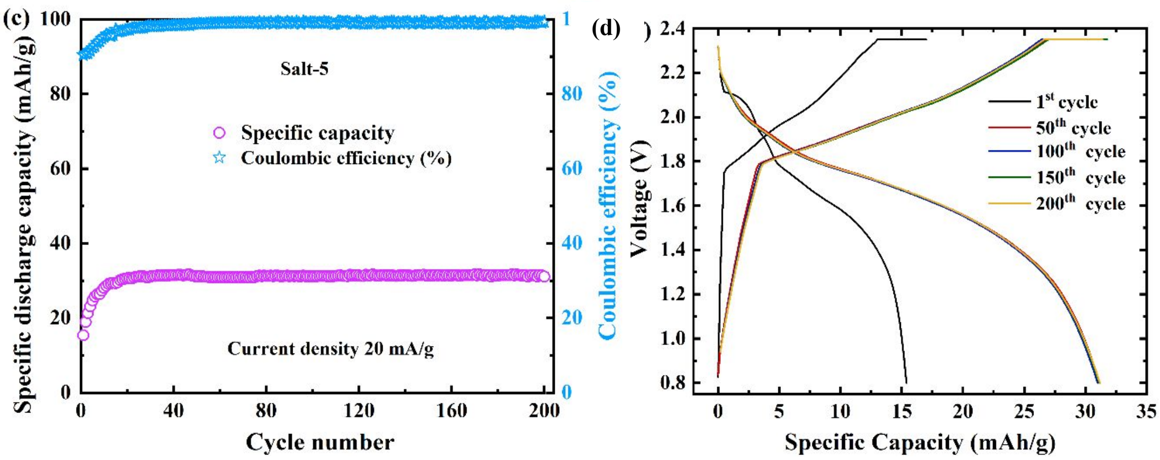

Figure S10. a) Charge-discharge curves of 1st cycle of all cells from Figure 6d of the main manuscript, b) absolute cell capacity versus cycle number for full cells with six different salt-based SPEs and c) specific discharge capacity and Coulombic efficiency versus cycle number for the salt-5 electrolyte up to 200 cycles and d) charge-discharge curves in the 1st, 50th, 100th, 150th and 200th cycle corresponding to Figure c).

Full cells were prepared using different SPEs, with spherical graphite (SpG) as the active material. Table S10 presents the mass of the cathode materials, from which the mass of the active material for each cell was determined.

Table S10. Salt names, active material, cathode mass, their active material mass and electrolyte mass.

Salt	Active material	Cell stack mass (g)	Cathode mass (g)	Active material mass (g)	Active material mass (mg cm^{-2})	Electrolyte mass (g)
Salt-1	SpG	0.191	0.030	0.012	6.0	0.145
Salt-2	SpG	0.193	0.031	0.013	6.5	0.146
Salt-3	SpG	0.192	0.030	0.012	6.0	0.146
Salt-4	SpG	0.191	0.030	0.012	6.0	0.145
Salt-5	SpG	0.191	0.031	0.013	6.5	0.144
Salt-6	SpG	0.192	0.030	0.012	6.0	0.146
Salt-5	SpG	0.191	0.030	0.012	6.0	0.145

Calculation for specific discharge capacity:

Given that for sample 1 (Salt-1):

- Total cathode weight (including current collector): $m_{\text{cathode,total}} = 0.030\,\text{g}$
- Current collector weight: $m_{\text{CC}} = 0.015\,\text{g}$
- Active material fraction: 80 % = 0.80
- Cathode area = 2 cm²

Step 1: Net cathode mass (without current collector)

$$m_{\text{cathode,net}} = m_{\text{cathode,total}} - m_{\text{CC}} \;\ldots\ldots\ldots\ldots\ldots\ldots(3)$$

$$m_{cathode,net} = 0.030 - 0.015 = 0.015\,\text{g} \;\ldots\ldots\ldots\ldots(4)$$

Step 2: Active material mass

$$m_{\text{active}} = m_{\text{cathode,net}} \times \text{ active material fraction } \ldots\ldots\ldots\ldots(5)$$

$$m_{active} = 0.015 \times 0.80 = 0.012\,\text{g} \;\ldots\ldots\ldots\ldots\ldots\ldots(6)$$

Specific capacity (based on active material):

$$\text{C}_{\text{sp}}\ (\text{mAh/g}) = \frac{Q\ (mAh)}{m_{active}}\ (\text{g}) \;\ldots\ldots\ldots\ldots(7)$$

Measured discharge capacity of the cell: Q (mAh) = ~0.42 mAh

$$\text{C}_{\text{sp}} = \frac{0.42\ \text{mAh}}{0.012\text{g}} = 35\frac{\text{mAh}}{\text{g}} \;\ldots\ldots\ldots\ldots(8)$$

Calculation for energy density:

The energy density was calculated by integrating the discharge voltage–capacity curve and normalizing the obtained energy to the combined mass of the cathode active material and the electrolyte. The energy density was calculated using the following equation:

$$\text{E}\left(\frac{\text{Wh}}{\text{kg}}\right) = \int \frac{\text{VdQ}}{m_{\text{cathode,active}} + m_{\text{electrolyte}}} \;\ldots\ldots\ldots\ldots\ldots(9)$$

where $\int$VdQ represents the area under the discharge curve, corresponding to the total discharge energy of the cell. The area under the discharge curve was determined using Origin software. For example, sample-1 (Salt-1), the integrated discharge energy is 6.70×10^{-4} Wh.

Area under the discharge curve = 6.70×10^{-4} Wh

The total active mass used for normalization is:

$$m_{\text{cathode,active}} + m_{\text{electrolyte}} = 0.012\,\text{g} + 0.145\,\text{g} = 1.57 \times 10^{-4}\text{kg} \;\ldots\ldots\ldots(10)$$

yielding an energy density of:

$$\mathrm{E} = 4.27 \text{ Wh kg}^{-1}$$

Coulombic efficiency calculation:

$$\mathrm{CE}\ (\%) = \frac{Q_{\text{discharge}}}{Q_{\text{charge}}} \times 100\ \% \text{.........}(11)$$

For sample-1 (Salt-1) 75th cycle efficiency, $\mathrm{CE}\ (\%) = \frac{34.85842}{34.9856} \times 100\ \% = 99.636\ \% \text{..}(12)$

S.3.7. Anode Surface Characterization

Pouch cell fabrication: Pouch cells with an active area of 3.5 cm^2 were assembled following a procedure similar to coin cell fabrication. After casting the solid polymer electrolyte onto the Al foil, the cathode of the desired size was attached, and the resulting assembly was cut to the required dimensions. Tab connections were then fixed to the electrodes. The cell components were subsequently stacked inside the pouch casing and vacuum-sealed to ensure a stable structure. Detailed procedures for pouch cell assembly can be found in the literature [4].

To investigate the anode surface, the pouch cell was cycled for 20 cycles at a current density of 20 mA g^{-1}. The cell was then carefully opened and disassembled to retrieve the anode. The extracted anode was cut into pieces of approximately 2 × 2 cm^2 for SEM and EDX analyses. A photographic image of the sample is shown in Figure S11a. Low and high-magnification SEM images of the bright and dark regions are presented in Figure S11b and S11c-d, respectively, and the corresponding impurity information is summarized in Table S11.

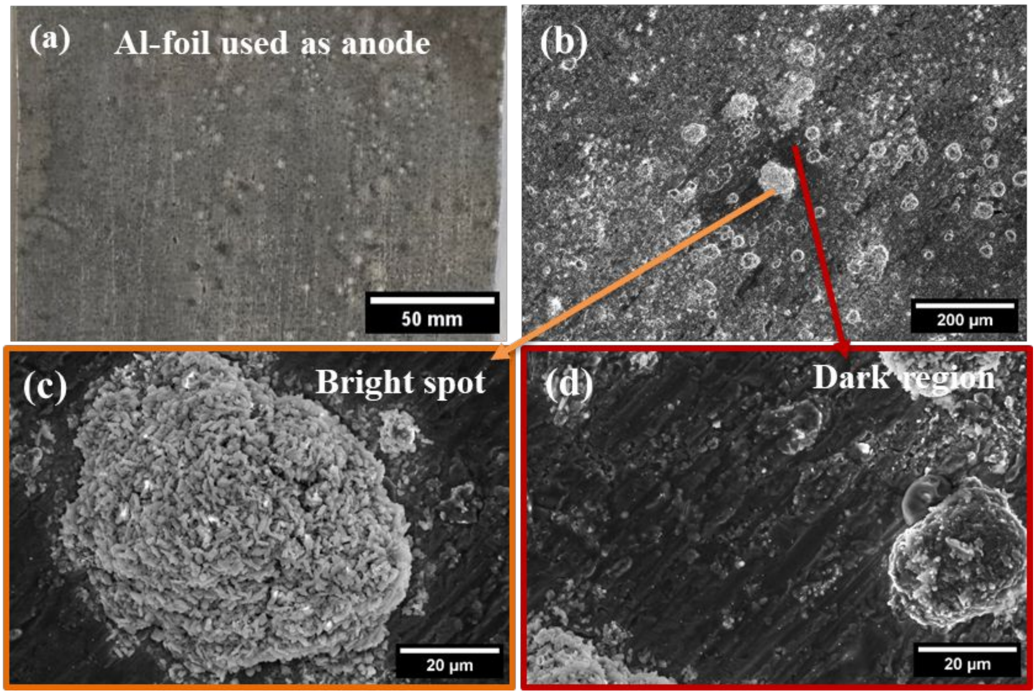

Figure S11. Photographic and SEM images: (a) Photographic image of Al foil anode after cycling and (b) low magnification SEM image of bright spot and dark region (c) high magnification SEM image of bright spot and (d) high resolution SEM images of dark region.

Table S11. Elemental composition of pristine and cycled Al foil determined by EDX, showing the detected elements and their relative contents.

**Pristine Al foil**			
**Element**	**Wt.-%**	**Atom %**	**Error %**
Al	95.55	94.23	13.19
O	1.86	3.09	16.65
Mg	1.50	1.64	14.75
Si	1.10	1.04	6.67
**Al foil used as anode**			
Al	80.50	72.15	11.30
O	16.89	25.53	15.46
Mg	1.35	1.34	12.90
Si	0.65	0.56	8.97
Cl	0.62	0.42	6.41

S.3.6. Cost-benefit analysis

The purities, prices, and chemical performances of various $AlCl_3$ salts are summarized in Table S12.

Table S12. Different purities of $AlCl_3$ salts and their electrochemical performance at a glance.

$AlCl_3$	Purity (%)	$AlCl_3$ price (€ g^{-1})	Conductivity (mS cm^{-1})	Capacity (mAh g^{-1})	Energy density (Wh kg^{-1})	CE (%)
Salt-1	99.999	6.198	0.21±0.02	34.86	4.27	99.64
Salt-2	99.999	5.86	0.19±0.01	30.58	3.51	98.18
Salt-3	99.99	2.18	0.21±0.00	28.71	3.33	97.94
Salt-4	99	0.47	0.21±0.00	43.50	4.97	97.94
Salt-5	99	0.027	0.21±0.01	32.20	4.24	98.99
Salt-6	98	0.36	0.21±0.01	33.55	4.49	98.26

The cost of accessories and other materials required to prepare an AlB coin cell was estimated individually, and the prices are summarized in Table S13.

Table S13. Accessories and other materials cost per cell of AIB

Casing + Spacer cost (€)	Al foil cost (€)	Polyimide foil cost (€)	Graphite cost (€)	Binder cost (€)	Total cost (€)
0.36	0.000345	0.0094	0.000996	0.000119	0.37086

References

[1] O. M. Leung, T. Schoetz, T. Prodromakis, C. P. De Leon, Progress in electrolytes for rechargeable aluminium batteries, Journal of the Electrochemical Society 168 (2021) 056509. doi:10.1149/1945-7111/abfb36.

[2] A. Mohammad, T. Köhler, S. Biswas, H. Stöcker, D. C. Meyer, A fexible solid-state ionic polymer electrolyte for application in aluminum batteries, ACS Applied Energy Materials 6 (2023) 2914–2923. doi:10.1021/acsaem.2c03906.

[3] Köhler, T., Mehner, E., Hanzig, J., Gärtner, G., Stöcker, H., Leisegang, T., & Meyer, D. C.. Real structure influencing the hydrogen defect chemistry in congruent $LiNbO_3$ and $LiTaO_3$. Journal of Solid-State Chemistry, 244 ((2016), 108-115.

[4] Rahman, M. M., Rana, M. J., Biswas, S., Mohammad, A., Stöcker, H., & Meyer, D. C. Temperature effects on the electrochemical behavior of graphite cathodes for aluminium-polymer batteries. Journal of Power Sources, 641 (2025), 236776.
